# The radioenhancement potential of Schiff base derived copper (II) compounds against lung carcinoma *in vitro*

**DOI:** 10.1371/journal.pone.0253553

**Published:** 2021-06-18

**Authors:** Gohar Tsakanova, Ani Stepanyan, Elina Arakelova, Violetta Ayvazyan, Vahan Tonoyan, Arsen Arakelyan, Guido Hildebrandt, Elisabeth Schültke

**Affiliations:** 1 Institute of Molecular Biology NAS RA, Yerevan, Armenia; 2 CANDLE Synchrotron Research Institute, Yerevan, Armenia; 3 Department of Radiooncology, Rostock University Medical Center, Rostock, Germany; Waterford Institute of Technology, IRELAND

## Abstract

For the last years, copper complexes have been intensively implicated in biomedical research as components of cancer treatment. Herewith, we provide highlights of the synthesis, physical measurements, structural characterization of the newly developed Cu(II) chelates of Schiff Bases, Cu(Picolinyl-L-Tryptopahanate)_2_, Cu(Picolinyl-L-Tyrosinate)_2_, Cu(Isonicotinyl-L-Tyrosinate)_2_, Cu(Picolinyl-L-Phenylalaninate)_2_, Cu(Nicotinyl-L-Phenylalaninate)_2_, Cu(Isonicotinyl-L-Phenylalaninate)_2_, and their radioenhancement capacity at kV and MV ranges of irradiation of human lung carcinoma epithelial cells *in vitro*. The methods of cell growth, viability and proliferation were used. All compounds exerted very potent radioenhancer capacities in the irradiated lung carcinoma cells at both kV and MV ranges in a 100 μM concentration. At a concentration of 10 μM, only Cu(Picolinyl-L-Tyrosinate)_2_, Cu(Isonicotinyl-L-Tyrosinate)_2_, Cu(Picolinyl-L-Phenylalaninate)_2_ possessed radioenhancer properties at kV and MV ranges. Cu(Picolinyl-L-Tryptophanate)_2_ showed radioenhancer properties only at kV range. Cu(Nicotinyl-L-Phenylalaninate)_2_ and Cu(Isonicotinyl-L-Phenylalaninate)_2_ showed remarkable radioenhancer activity only at MV range. All compounds acted in dose-dependent manner at both tested energy ranges. These copper (II) compounds, in combination with 1 Gy irradiation at either 120 kV or 6 MV, are more efficient at delaying cell growth of lung cancer cells and at reducing cell viability *in vitro* than the irradiation administered alone. Thus, we have demonstrated that the studied copper compounds have a good potential for radioenhancement.

## Introduction

**Fig 1:**
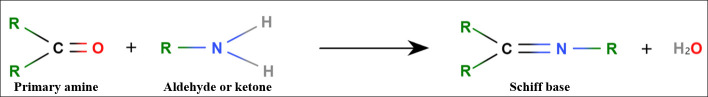
General scheme of synthesis of Schiff bases (modified from [27]).

Schiff base copper complexes are azimethine group (-CH = N-) containing organic compounds generated by the condensation of primary amines and aldehydes or ketones, which contain a copper ion in their structure (Fig 1) [1]. Potentially, these complexes have a wide range of applications due to their catalytic, antimicrobial, regulatory, pharmacological as well as antiradical and antioxidant features [2–7]. *In vitro*, Schiff base copper complexes have been proven to possess a beneficial role in the treatment of different pathological conditions, including bacterial [7–9], viral [7] and fungal [7–9] infections, and the treatment of diabetes mellitus [2]. Potentially, there might be also a role in the treatment of cancer [2, 7, 10–15]. For the last years, copper complexes have been intensively studied for their tumoricidal features as components of cancer treatment [16–25]. It has been shown that aliphatic aldehydes in the structure of Schiff bases destabilize, whereas aromatic aldehydes stabilize the molecule of Schiff bases due to the conjugation system [26, 27].

Although some properties of Schiff base copper complexes are already well known, they still are considered a novel class of molecules with expanding fields of applications in the design of new therapeutic agents [16–25] offering a huge potential to synthesize new Schiff base-derived complexes with more advanced features.

According to data compiled by the World Health Organization, lung cancer is the most common cancer worldwide, accounting for approx. 2.1 million new cases and 1.8 million deaths in 2018 [28], more than breast, colon and prostate cancers combined [29]. Despite an imposing number of treatment strategies developed over the past 50 years, including new immunotherapies and personalized medicine [29], the mortality rate of patients with lung cancer is still very high. A high percentage of patients die within one year of being diagnosed [30]. Hence, there is an urgent need to find more efficient strategies to increase the success rate of lung cancer treatment.

In our previous work, we reported about the synthesis, structural characterization and toxicity of several Schiff bases and their copper complexes. It has been found that these compounds demonstrate high antiradical/antioxidant activity, stimulate the activities of enzymes of endogenous antioxidant defense systems and regulate the levels of biomarkers of the organism’s immune system, demonstrating anti-inflammatory and immunomodulatory effects [31–35].

In this paper, we provide highlights of the synthesis, physical measurements, structural characterization of the newly developed Cu(II) chelates of Schiff Bases, Cu(II) (Picolinyl-L-Tryptopahanate)_2_, Cu(II) (Picolinyl-L-Tyrosinate)_2_, Cu(II) (Isonicotinyl-L-Tyrosinate)_2_, Cu(II) (Picolinyl-L-Phenylalaninate)_2_, Cu(II) (Nicotinyl-L-Phenylalaninate)_2_, Cu(II) (Isonicotinyl-L-Phenylalaninate)_2_, as well as their cytotoxic activity against human lung carcinoma epithelial cells *in vitro*. We hypothesized that, in addition to known cytotoxic features, some of these compounds may be suitable as radioenhancing agents in cancer therapy.

## Materials and methods

### Chemical synthesis of Schiff base copper (II) complexes

The synthesis and characterization of Schiff base copper complexes have been carried out according to previously described methods [31]. All synthesis procedures were performed under normal atmospheric conditions.

#### Chemical synthesis of initial Schiff bases

The first step was the synthesis of Schiff bases, Picolinyl-L-Tryptopahanate, Picolinyl-L-Tyrosinate, Isonicotinyl-L-Tyrosinate, Picolinyl-L-Phenylalaninate, Nicotinyl-L-Phenylalaninate and Isonicotinyl-L-Phenylalaninate. For this purpose, amino acids, L-Tryptophan, L-Tyrosine and L-Phenylalanine were condensated with 2-, 3- or 4-pyridinecarboxaldehydes (Picolinaldehides, Nicotinaldehydes or Isonicotinaldehydes) in presence of alcohol. A 5°C–25°C temperature range and molar ratio of 1:1 were maintained. Briefly, 10 mM of L-Tryptophan, L-Tyrosine or L-Phenylalanine solutions were prepared in 100 mL of alcohol solution (methanol), containing 10 mM KOH or 20 mM NaOH with subsequent permanent stirring for 1 hour (until complete solution of the amino acid) under the conditions of dry nitrogen and a temperature of 18°C–20°C. Then, 10 mM of 2-, 3- or 4-pyridincarboxyaldehyde dissolved in 50 mL of ethanol was added with subsequent stirring and reflux for 2 hours at 50°C until getting a yellow colored solution corresponding to Schiff base formation.

#### Chemical synthesis of Schiff base copper (II) complexes

After the synthesis of initial Schiff bases, the second step was the synthesis of copper complexes, for which the initial Schiff bases served as ligands. The synthesis was performed in methanol at 20±2°C using KOH and copper (II) acetate. Complex formation was carried out in a reaction medium without preliminary Schiff base isolation. Briefly, 60 mL methanol and 5 mM of monohydrate copper acetate, Cu(CH_3_COO)_2_×H_2_O in the form of blue-green small crystals was added to the previously obtained solution of Schiff base (Picolinyl-L-Tryptopahanate, Picolinyl-L-Tyrosinate, Isonicotinyl-L-Tyrosinate, Picolinyl-L-Phenylalaninate, Nicotinyl-L-Phenylalaninate or Isonicotinyl-L-Phenylalaninate) with subsequent stirring for 3 hours until getting color change corresponding to copper complex formation.

For isolation of copper complexes, the solvent was partially evaporated with the subsequent settling, centrifugation, re-crystallization, and vacuum drying. The reduction in solution volume was implemented using a vacuum rotary evaporator to obtain 75%-80% of evaporation, after which the remainder was kept in dark for 12 hours with subsequent centrifugation. After repeated centrifugation and supernatant discharge, the resulting precipitate was washed off twice: firs with deionized water followed by ethanol, then by dimethyl ether using 5 mL wash-off liquid each time. The formed copper complexes were then dried under vacuum conditions at pressure of 2–3 mm Hg and 70°C.

L-tryptophan, L-Tyrosine, L-Phenylalanine, KOH, NaOH, copper (II) acetate, methanol, as well as 2-, 3- and 4-pyridinecarboxaldehydes were purchased from Sigma Aldrich (Sigma Aldrich Co. LLC, USA).

#### Characterization of initial Schiff bases and their copper (II) complexes

For characterization of Schiff bases and their copper complexes, infrared absorbance spectra were obtained in the range of 400–4000 cm^-1^ in Vaseline oil on KBr plates using a Spectrometer IR75 (Carl Zeiss, Jena). The elemental analyses for K, Na, C, H and N were performed by combustion in a pure oxygen environment using a PerkinElmer 2400 Series II CHNS/O Elemental Analyzer (PerkinElmer, USA). The nuclear magnetic resonance (NMR) spectra were obtained in D_2_O and CD_3_OD on a Varian 300 MHz Spectrometer. The comparative investigation on UV/Vis spectral analysis of absorbance of initial Schiff bases and their Cu complexes was performed in the range of 200–900 nm using a Specord M40 Spectrometer (Carl Zeiss, Jena). The cuvette thickness was 1.02 mm, and the concentration of studied substances ranged from 2×10^−4^ to 5×10^−4^ mol/L in methanol.

The thermo-stability assessment of synthesized copper complexes was conducted by exposure to thermal effects during a period of 2 hours at 60°С-100°С temperature range. IR absorbance spectra were studied prior to and after heating. To heat the samples, special cuvettes warmed via the ultra-thermostat were used. The spectra were recorded every 30 min.

The solubility assessment of copper complexes was carried out according to standard test method protocol [36]. The three-dimensional (3D) structures of the synthesized Schiff bases and their copper complexes have been predicted *in silico* using MolView program [37, 38].

### Experimental design of the radiobiology study

**Fig 2:**
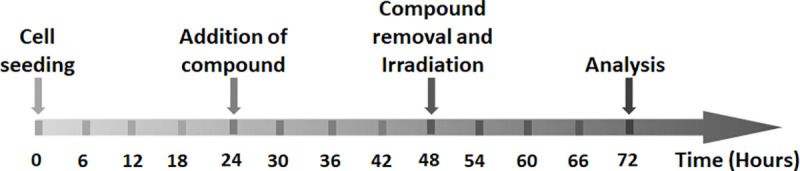
Schematic flow diagram of the experimental study design.

Exponentially growing A549 human lung cancer cells were sub-cultured into 12- or 96-well culture plates. The colloid solutions of Schiff base copper complexes were added at 24 hours after seeding and were removed after 24 hours of exposure, just prior to irradiation (Fig 2). To analyze the effects of ionizing radiation at kV and MV ranges on the cells, we conducted viability and proliferation tests at 24 hours after irradiation and cumulative cell growth counts at 3, 4 and 8 days after cell seeding. Naïve cells and non-irradiated cells treated with Schiff base copper complexes served as controls.

### Cell culture

The experiments were performed using A549 human lung carcinoma epithelial cells obtained from American Type Culture Collection (ATCC^®^ CCL-185™). The cell lines were cultured in T75 and T175 flasks in Dulbecco’s modified Eagle’s medium (DMEM; Lonza BioWhittaker, Verviers, Belgium) supplemented with 10% fetal bovine serum (FBS) and 1% penicillin/streptomycin (P/S) with a total volume of 5 mL and 50 mL, respectively, under standard incubation conditions (37°C, 5% CO_2_ humidified atmosphere). To harvest the cells, the cultures were washed with 1×PBS and detached from the bottom of the flask using 3 mL of 0.25% (w/v) Trypsin-0.53 mM EDTA solution for T75 flasks and 20 mL of 0.05% (w/v) Trypsin-0.53 mM EDTA solution for T175 flasks.

### Preparation of the solutions of Schiff base copper complexes

The stock solution of each Schiff base copper complex was prepared with the initial concentrations of 0.1 mM and 1 mM, the final concentration in the medium was 10 μM and 100 μM, respectively. The compounds are insoluble in both water and PBS, but soluble in Dimethyl sulfoxide (DMSO). However, considering that DMSO itself is cytotoxic, the compounds were suspended in 1×PBS and used in colloid form.

### Irradiation

Irradiation was performed at room temperature, using two different X-ray sources. For irradiation at kV range, an Xstrahl 200 system (Xstrahl Ltd., Surrey, United Kingdom) was used at 120 kV with a 50 cm FSD (focus-to-skin distance) closed clinical applicator and a 5 mm Aluminium HVL (half value layer) filter. For irradiation at MV range, a Versa HD linear accelerator (Elekta Oncology Systems, Crawley, UK) was used at 6 MV. The irradiation of cells was performed with a dose of 1 Gy at 48 hours after seeding and 24 hours exposure to the compound tested. After irradiation, the cells were returned to the incubator. The parameters of kV and MV irradiation are provided in Table 1.

**Table 1:** Parameters for X-ray irradiation.

Parameters	kV irradiation	MV irradiation
**Target dose**	1 Gy	1 Gy
**Dose rate**	50 MU/min	91.2 MU/min
**Radiation delivery time**	2.07 min	< 1 min
**Source**	Xstrahl200	VersaHD linac
**Energy**	120 kV	6 MV
**Field size**	20 cm x 20 cm	40 cm x 40 cm

### Cumulative cell growth

**Fig 3:**
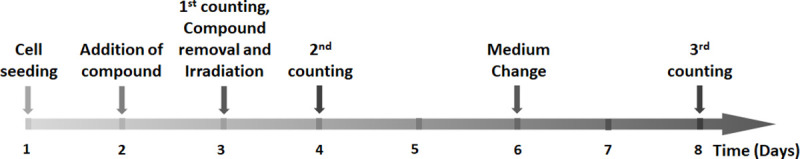
Schematic of the toxicity test experiment.

To calculate the cumulative cell growth, cell counting was performed at three different time points: immediately before irradiation, 24 hours and 5 days after irradiation using an automated cell counter (Coulter Z2, Beckmann Coulter GmbH, Krefeld, Germany) (Fig 3). For this experiment, the cells were seeded into 12-well culture plates at a density of 20×10^3^ cells per well in a total volume of 1 mL.

### Cell viability assay

Cell viability was tested using water-soluble tetrazolium 1 (WST-1) Cell Proliferation Reagent (Roche Deutschland GmbH, Mannheim, Germany). The cells were seeded into 96-well culture plates in sextuplicate at a density of 5×10^2^ cells per well. Following cellular adhesion (24 hours after seeding), the copper compounds were added for 24 hours. The cells were irradiated in fresh medium after removal of compound. The WST-1 Cell Proliferation Reagent was added 24 hours after irradiation and spectrophotometric measurement (absorbance) was conducted after 2 hours of incubation using Anthos Zenyth 340r plate reader (Anthos Mikrosysteme GmbH, Krefeld, Germany) at an excitation wavelength of 450 nm with a reference wavelength of 620 nm (Fig 3).

### Cell proliferation assay

The 5-bromo-2’-deoxyuridine (BrdU) cell proliferation ELISA colorimetric test (Cell Proliferation ELISA, Roche Applied Science, Mannheim, Germany) was used to determine the changes in cell proliferation rate after irradiation. The cells were seeded into 96-well culture plates in sextuplicate at a density of 1×10^3^ cells per well. Following cellular adhesion (24 hours after seeding), the copper compounds were added for 24 hours, then removed and the cells were irradiated in freshly added growth medium. The BrdU enzyme linked immunosorbent analysis (ELISA) was performed 24 hours after irradiation (Fig 2) according to the manufacturer’s instructions, with 2 hours of BrdU labeling. The absorbance of the samples was measured using Anthos Zenyth 340r reader at an excitation wavelength of 340 nm with a reference wavelength of 492 nm.

### Statistical analysis

Statistical analysis was performed using Graphpad Prism 5.0 software (GraphPad Software Inc., USA). For cell counts, Two-way ANOVA with repeated measurements and Bonferroni post-tests was used. The statistical analyses for WST-1 and BrdU tests were done using Ordinary Two-way ANOVA followed by Bonferroni post-tests.

All error bars indicate the standard error of the mean (SEM) for at least three repeats in the figures. Significant differences were presumed when *P* < 0.05. Particularly, *P* < 0.05 values are marked as “*”, *P* < 0.01 values are marked as “**”, *P* < 0.001 values are marked as “***”, and *P* < 0.0001 values are marked as “****”.

## Results

### Chemical synthesis of Schiff base copper compounds and their characterization

The Schiff base synthesis of L-tryptophan, L-tyrosine and L-phenylalanine with potassium or sodium salts, respectively, yielded target products up to 85%, 80%, 82%, 58%, 62% and 73% for Cu(Picolinyl-L-Tryptophanate)_2_, Cu(Picolinyl-L-Tyrosinate)_2_, Cu(Isonicotinly-L-Tyrosinate)_2_, Cu(Picolinyl-L-Phenylalaninate)_2_, Cu(Nicotinyl-L-Phenylalaninate)_2_ and Cu(Isonicotinyl-L-Phenilalaninate)_2_, respectively. The Schiff bases were isolated as brown solids for Cu(Picolinyl-L-Tryptophanate)_2_, Cu(Picolinyl-L-Tyrosinate)_2_ and Cu(Picolinyl-L-Phenylalaninate)_2_, yellow solid for Cu(Isonicotinly-L-Tyrosinate)_2_, and a green solid for Cu(Nicotinyl-L-Phenylalaninate)_2_ and Cu(Isonicotinyl-L-Phenilalaninate)_2_.

The results of the elemental analysis of the synthesized Schiff bases and their copper (II) complexes are presented in Table 2. Based on these data, empirical formulas were proposed and the molecular weight of the synthesized Schiff bases and their copper (II) complexes was calculated (Table 3).

**Table 2:** Elemental analysis of synthesized Schiff bases and their copper (II) complexes.

Nanocompound	Na/K/Cu (%)		C (%)		H (%)		N (%)	
	Calc.	Obs.	Calc.	Obs.	Calc.	Obs.	Calc.	Obs.
**K(Picolinyl-L-Tryptophanate)**	11.80	11.62	61.61	61.88	4.26	4.63	12.68	12.53
**Cu(Picolinyl-L-Tryptophanate)**_**2**_	9.80	9.34	63.00	63.28	4.35	4.76	12.97	12.81
**Na(Picolinyl-L-Tyrosinate)**	7.87	8.09	61.64	61.32	4.48	4.82	9.59	9.78
**Cu(Picolinyl-L-Tyrosinate)**_**2**_	10.55	10.69	59.85	59.43	4.35	4.91	9.31	9.12
**Na(Isonicotinly-L-Tyrosinate)**	7.87	7.56	61.64	61.37	4.48	4.85	9.59	9.81
**Cu(Isonicotinly-L-Tyrosinate)**_**2**_	10.55	11.09	59.85	59.62	4.35	4.87	9.31	9.42
**Na(Picolinyl-L-Phenylalaninate)**	8.33	8.54	65.21	64.88	4.74	4.37	10.14	13.43
**Cu(Picolinyl-L-Phenylalaninate)**_**2**_	10.80	10.40	61.27	62.60	4.80	4.7	9.53	9.38
**Na(Nicotinly-L-Phenylalaninate)**	8.32	8.23	65.21	64.93	7.74	5.08	10.14	10.32
**Cu(Nicotinly-L-Phenylalaninate)**_**2**_	16.91	16.53	54.32	54.72	4.29	4.65	7.45	7.21
**Na(Isonicotinyl-L-Phenilalaninate)**	8.32	8.12	65.21	65.42	4.74	4.42	10.14	9.91
**Cu(Isonicotinyl-L-Phenilalaninate)**_**2**_	11.15	11.03	63.20	62.82	4.60	4.83	11.22	10.97

*Calc*.: *calculated; Obs*.: *observed*.

**Table 3:** Empirical formulas and molecular weights of the synthesized Schiff bases and their copper (II) complexes.

Nanocompound	General formula	Molecular weight
**K(Picolinyl-L-Tryptophanate)**	C_17_H_14_N_3_O_2_K	331.41
**Cu(Picolinyl-L-Tryptophanate)**_**2**_	C_34_H_28_N_6_O_4_Cu	648.17
**(Picolinyl-L-Tyrosinate)**	C_15_H_13_N_2_O_3_Na	292.27
**Cu(Picolinyl-L-Tyrosinate)**_**2**_	C_30_H_26_N_4_O_6_Cu	602.1
**(Isonicotinly-L-Tyrosinate)**	C_15_H_13_N_2_O_3_Na	292.27
**Cu(Isonicotinly-L-Tyrosinate)**_**2**_	C_30_H_26_N_4_O_6_Cu	602.1
**Na(Picolinyl-L-Phenylalaninate)**	C_15_H_13_N_2_O_2_Na	276.27
**Cu(Picolinyl-L-Phenylalaninate)**_**2**_	C_30_H_28_N_4_O_5_Cu	588.12
**(Nicotinly-L-Phenylalaninate)**	C_15_H_13_N_2_O_2_Na	276.27
**Cu(Nicotinly-L-Phenylalaninate)**_**2**_	C_17_H_16_N_2_O_4_Cu	375.87
**(Isonicotinyl-L-Phenilalaninate)**	C_15_H_13_N_2_O_2_Na	276.27
**Cu(Isonicotinyl-L-Phenilalaninate)**_**2**_	(C_15_H_13_N_2_O_2_)_2_.Cu	570.11

The analysis of IR absorbance spectra revealed that upon the formation of copper (II) complexes a shift of IR absorbance spectra, compared to the Schiff bases, is observed. Particularly, the analysis of IR spectra of synthesized compounds showed the presence of a C = N bond at the frequencies presented in Table 4. Further, upon formation of the copper (II) complexes, the valence deviations of the C = N bond is shifted towards the low frequencies compared to Schiff bases. Moreover, the coordination bond Cu. . . .N results in widening and shift of valence deviations (C = N) towards the low frequencies and this band is partially overlapped by (CО^-^)_as_ band of valence deviations.

**Table 4:** IR absorbance spectra of synthesized Schiff bases and their copper (II) complexes.

Nanocompound	ν(C = N), cm^-1^	ν_as_(CO^-^), cm^-1^	ν_s_(CO^-^), cm^-1^	ν(C-N), cm^-1^
**K(Picolinyl-L-Tryptophanate)**	1642	1597	1405	1086
**Cu(Picolinyl-L-Tryptophanate)**_**2**_	1631	1606	1363	1073
**(Picolinyl-L-Tyrosinate)**	1656	1582	1405	1108
**Cu(Picolinyl-L-Tyrosinate)**_**2**_	1633	1587	1581	1101
**(Isonicotinly-L-Tyrosinate)**	1640	1586	1397	1109
**Cu(Isonicotinly-L-Tyrosinate)**_**2**_	1620	1581	1389	1103
**Na(Picolinyl-L-Phenylalaninate)**	1659	1607	1408	1086
**Cu(Picolinyl-L-Phenylalaninate)**_**2**_	1635	1605	1383	1075
**(Nicotinly-L-Phenylalaninate)**	1648	1613	1402	1084
**Cu(Nicotinly-L-Phenylalaninate)**_**2**_	1634	1602	1388	1073
**(Isonicotinyl-L-Phenilalaninate)**	1646	1611	1399	1088
**Cu(Isonicotinyl-L-Phenilalaninate)**_**2**_	1640	1609	1387	1080

The presence of a–HC = N–imine bond was confirmed by a signal in the range of 8.01 m.d. (singlet) in NMR ^1^Н spectrum for K(Picolinyl-L-Tryptophanate), 7.99 ppm (singlet) in NMR ^1^Н spectrum for Na(Picolinyl-L-Phenylalaninate), 7.8 m.d. (singlet) for Na(Nicotinyl-L-Phenylalaninate) and 7.68 m.d. (singlet) for Na(Isonicotinyl-L-Phenilalaninate), Na(Picolinyl-L-Tyrosinate), Na(Isonicotinly-L-Tyrosinate).

The IR absorbance spectra of Picolinyl-L-Tryptophanate show that upon the formation of this Schiff base, the valence deviation of the tryptophan indole ring (NH) was shifted from 3430 cm-1 to 3180–3190 cm^-1^ suggesting the intramolecular interaction of indole and pyridine rings. Then, upon the formation of its copper (II) complex, Cu(Picolinyl-L-Tryptophanate)_2,_ this band appeared in the area of 3250–3270 cm^-1^, which, in fact, is associated with the changes in conjugation linkage degree (C = N) with pyridine ring caused by coordination bond Cu. . . .N and resulting in changes of interactions between the pyridine and indole rings. Moreover, the analysis of NMR spectra (H^1^ and C^13^) demonstrated the presence of the signals of protons of the group ─СН_2_─СН─ and a proton of the group ─Ν═СН─. The presence of CH signal (duplet-duplets) of–СН_2_-СН- in the range of 4.1–4.5 ppm is characteristic for ^1^Н NMR spectra of studied Schiff bases.

In the IR absorbance spectra of Cu(Picolinyl-L-Tyrosinate)_2_ and Cu(Isonicotinyl-L-Tyrosinate)_2_ binding of alkali metal with the acidic residue of this amino acid caused a shift of ν bond (С = О) in from 1611 cm^-1^ to 1582 cm^-1^ due to re-arrangement of π electrons of the double bond (С = О).

Upon the formation of Picolinyl-L-Tyrosinate and Isonicotinyl-L-Tyrosinate, changes are observed in the area of ν (C-N) bond shifting the valence deviation C-N towards lower frequencies. Moreover, upon formation of the copper (II) complex, this band becomes wider with the frequencies of 1101 cm^-1^ and 1103 cm^-1^. These changes, as well as the presence of (Cu─О) bond in the frequency range of 400–450 cm^-1^, support the assumption that the obtained compounds are copper complexes.

For Picolinyl-L-Phenylalaninate, a powerful wide absorbance band was observed in the range of 1610 cm^-1^ due to valence deviations ν_as_ (С = О) of both the amino acid and pyridine rings of aldehyde. Moreover, valence deviations (C = O) of phenylalanine shifted to this range after substitution of hydrogen in acidic group СООН by sodium (СООNa), and the valence deviation band C = N characteristic for Schiff bases is observed in the range of 1645–1660 cm^-1^ as a shoulder at the strong band seen at 1600 cm^-1^. A strong band in the range of 1400±5 cm^-1^ corresponds to absorbance of ionized carboxyl group (Table 4). The spectroscopic data support the assumption that metal complexes contain two Schiff base ligands.

With the help of derivatography it was established that Cu(II) metal complexes have no melting points, as upon exposure to thermal effect they decompose in the temperature range from 180° С to 200° С. The comparison of IR-spectra of heated samples with those of the initial, naïve samples demonstrated that 2-hour heating caused no structural changes.

**Fig 4:**
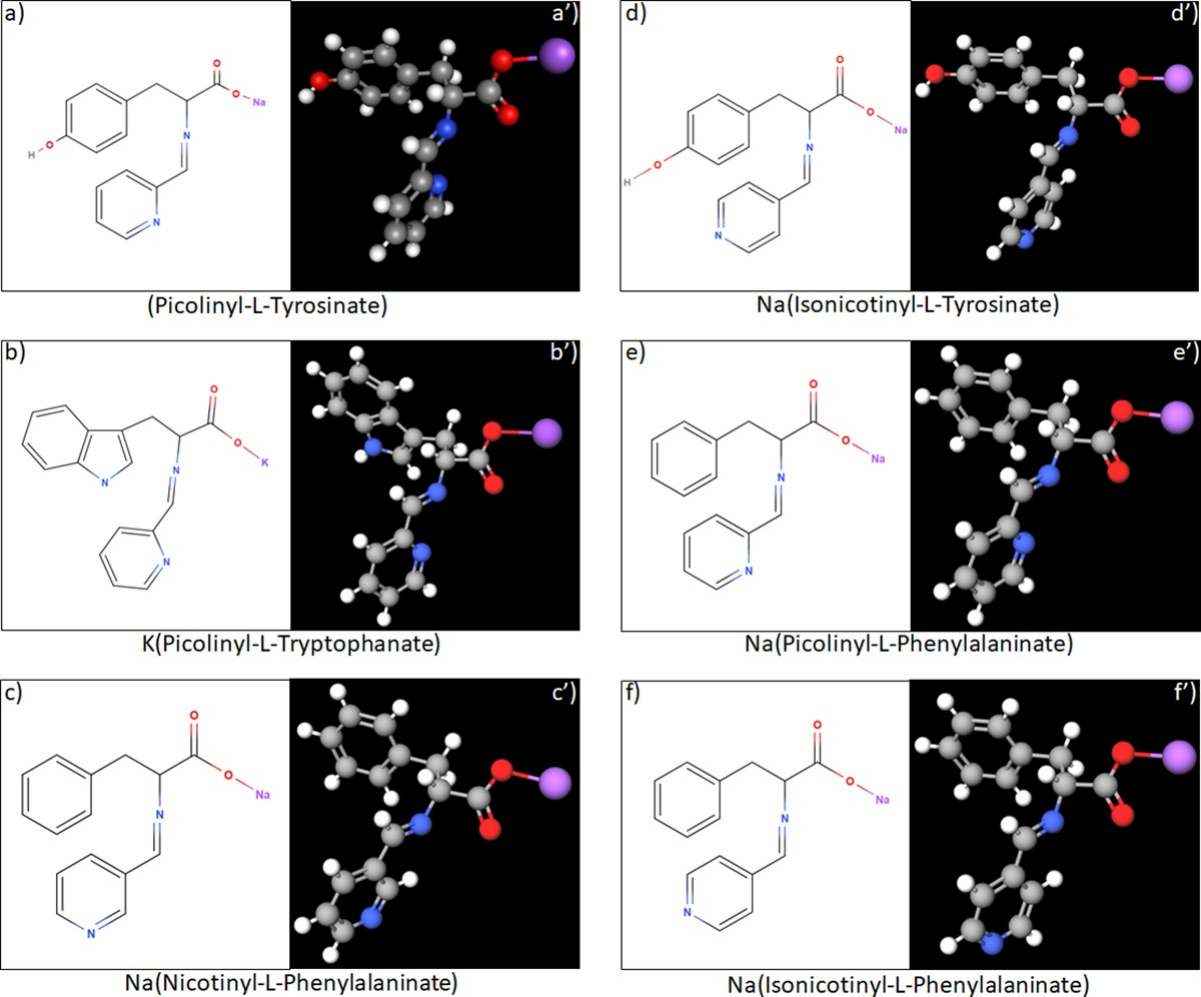
Proposed 2D structures (4a-4f) and *in silico* predicted 3D structures (4a’-4f’) of the synthesized Schiff bases. Grey color corresponds to carbon (C) atom, white color corresponds to hydrogen (H) atom, blue color corresponds to nitrogen (N) atom, red color corresponds to oxygen (O) atom, and purple color corresponds to sodium (Na) or potassium (K) atoms.

**Fig 5:**
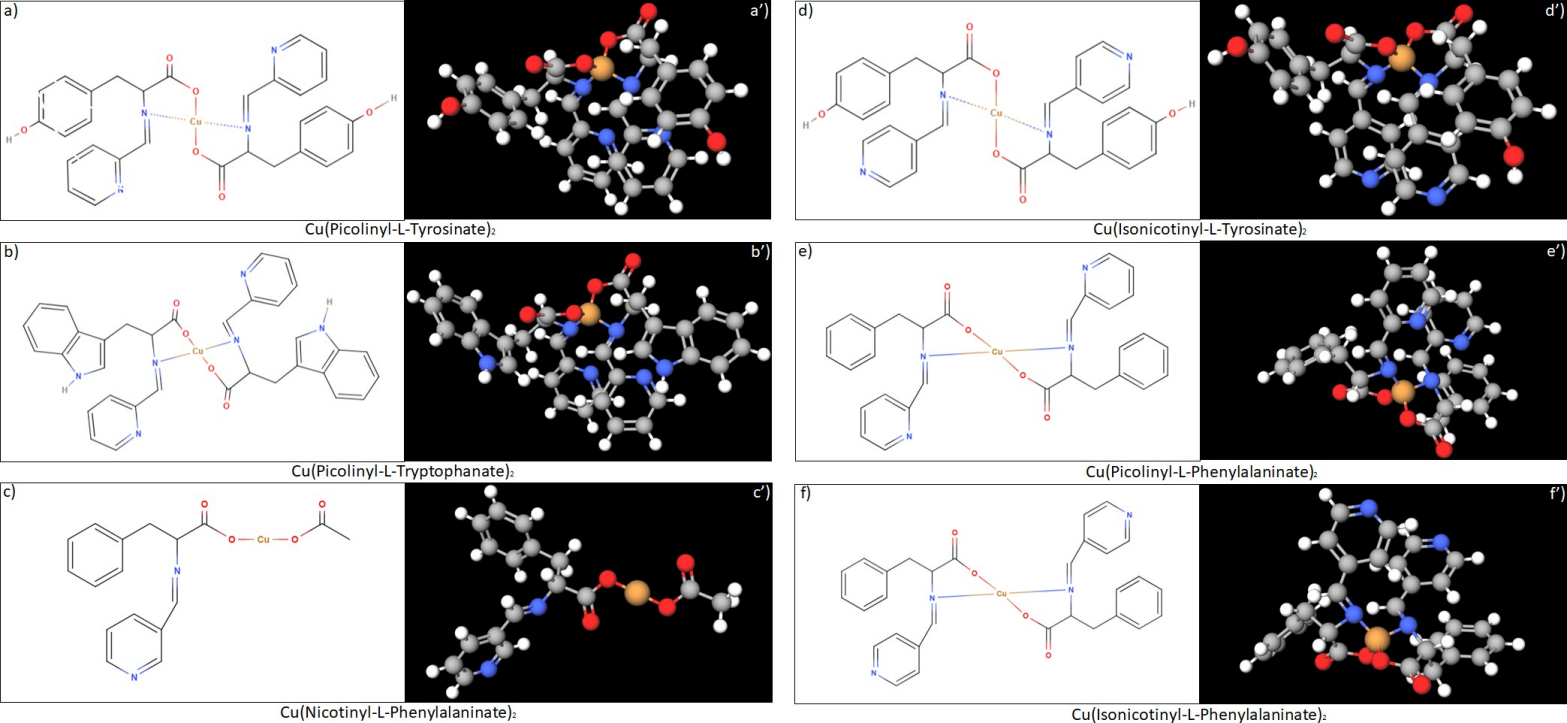
Proposed 2D structures (4a-4f) and *in silico* predicted 3D structures (4a’-4f’) of synthesized copper (II) complexes. Grey color corresponds to carbon (C) atom, white color corresponds to hydrogen (H) atom, blue color corresponds to nitrogen (N) atom, red color corresponds to oxygen (O) atom, and orange color corresponds to copper (Cu) atom.

The proposed structure of synthesized Schiff bases and their copper complexes are presented in Figs 4 and 5. Based on UV absorbance spectra (Table 2), upon complex formation with Cu(II) the structure of Schiff Bases undergoes almost no changes. However, the initial Schiff bases are soluble in water, in contrast to their copper complexes, which are insoluble in water but soluble in DMSO.

### Assessment of the effect of PBS on cell cultures

**Fig 6:**
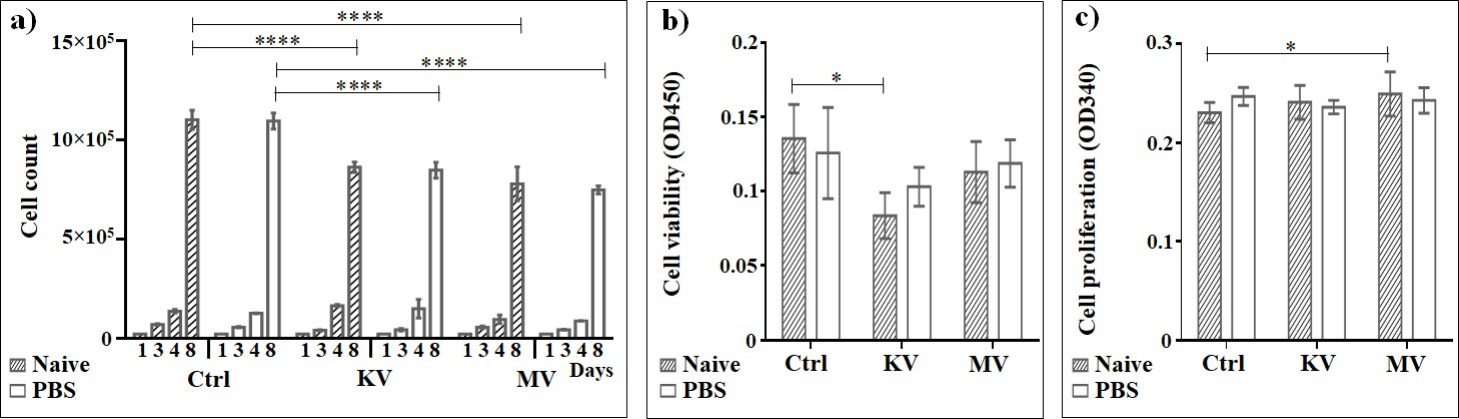
The effect of PBS without copper (II) complexes on A549 lung carcinoma epithelial cells, irradiated vs. non-irradiated at the kV or MV range. a) Cumulative cells growth; b) WST-1 cytotoxicity assay; c) BrdU cell proliferation assay. *The graphs show the mean ± standard error of the mean (M ± SEM)*. *P < 0.05; **P < 0.01; ***P < 0.*001*.

To analyze the cell growth of A549 lung carcinoma cells under different conditions of interest for treatment, with copper (II) compounds in the concentrations of 10 μM and 100 μM, cumulative cell numbers were counted on the 3rd (24 after treatment with copper (II) complexes, just before irradiation), 4th (24 hours after irradiation) and 8th days after cell seeding (Fig 6). The WST-1 cytotoxicity and BrdU cell proliferation tests were conducted on the 4th day of cell growth.

PBS, used as suspension medium for the copper (II) complexes, does not have any significant effect on cell growth (Fig 6A and S1 Table), viability (Fig 6B and S2 Table) and proliferation (Fig 6C and S3 Table) in any of the experimental groups (*P* > 0.05). With a factor of approx. 1.3 compared to non-irradiated cells, deceleration of the cell growth (between days four to eight after seeding) was slightly slower in the kV range, compared to a factor of 1.45 in the MV range (*P* < 0.001).

When comparing the cell viability between non-irradiated and irradiated cells in the WST-1 test, the only statistically significant decrease in cell viability (approx. 1.6 times) was seen after kV irradiation (Fig 6B and S2 Table). With regard to the cell proliferation assessed by the BrdU incorporation, a small, however statistically significant (*P* < 0.05) difference was found after irradiation of naïve cells at the MV range (1.08 times increase compared to non-irradiated naïve cells) (Fig 6C and S3 Table).

### Assessment of the effect of Cu(Picolinyl-L-Tyrosinate)_2_ on cells

Cu(Picolinyl-L-Tyrosinate)_2_ significantly decelerates the cell growth of the control non-irradiated cells at four days (3.44 and 9.8 times for 10 μM and 100 μM, respectively) and eight days (3.52 and 60 times for 10 μM and 100 μM, respectively). Thus, the compound is cytotoxic for the lung cancer cells.

**Fig 7:**
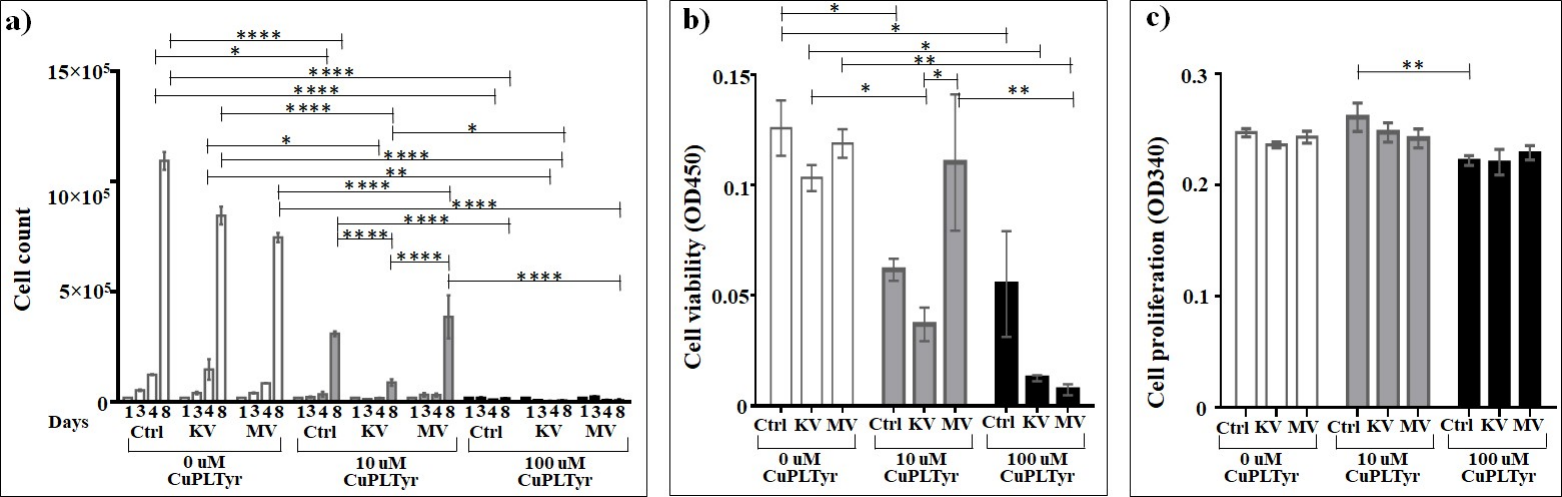
The effect of Cu(Picolinyl-L-Tyrosinate)_2_ on A549 lung carcinoma epithelial cells irradiated or non-irradiated with kV or MV irradiation. a) Cumulative cells growth; b) WST-1 cell viability assay; c) BrdU cell proliferation assay. *The graphs show the mean ± standard error of the mean (M ± SEM)*. *P < 0.05; **P < 0.01; ***P < 0.*001*.

Compared to non-irradiated controls, cell death is increased by treatment with this compound on day four (7.7 and 23 times for 10 μM and 100 μM, respectively) and day eight (9.43 and 105.2 times for 10 μM and 100 μM, respectively) when the cells are exposed to irradiation at the kV range, and only on day eight when the cells are exposed to irradiation at the MV range (1.93 and 83.73 times for 10 μM and 100 μM, respectively) (S4 Table). Compared to control non-irradiated treated cells, the addition of 10 μM of Cu(Picolinyl-L-Tyrosinate)_2_ 3.5 times decreased the growth of the cells irradiated at the kV range, while 1.25 times significantly accelerates the cell growth at MV range of irradiation. After irradiation at the MV range, 10 μM of this compound significantly decreased the cell growth on day eight after irradiation at kV range. The treatment with 100 μM of this compound significantly suppressed cell death on day eight (11.2 times with kV and 43.44 times with MV irradiation) (Fig 7A, S4 Table). Interesting here is that the increase in cell death is four times higher with MV as compared to kV irradiation.

Compared to irradiation alone, the addition of 10 μM Cu(Picolinyl-L-Tyrosinate)_2_ resulted in a 9.4 times significant (*P* < 0.0001) increase in cell death at the kV and a 2 times significant (*P* < 0.0001) increase at the MV range. For 100 μM, the significant enhancement in cell death was 105 times (*P* < 0.0001) at the kV and 84 times (*P* < 0.0001) at the MV range (based on cell counts day 8).

No statistically significant changes were seen as effect of Cu(Picolinyl-L-Tyrosinate)_2_ on the cell viability assessed by the WST-1 test, compared to irradiation alone. The addition of 10 μM Cu(Picolinyl-L-Tyrosinate)_2_ resulted in a 3 times significant (*P* < 0.05) decrease in cell viability at kV range and no statistically significant change (*P* > 0.005) at MV range, while the addition of 100 μM Cu(Picolinyl-L-Tyrosinate)_2_ resulted in a 8 times significant (*P* < 0.05) decrease in cell viability at the kV range and a 17 times significant (*P* < 0.01) decrease at the MV range. Meanwhile, the addition of 10 μM Cu(Picolinyl-L-Tyrosinate)_2_ decrease the cell viability 3 times (*P* < 0.05) more intense at kV range than at MV range, while no statistically significant difference (*P* > 0.05) is observed between these two ranges in case of 100 μM (Fig 7B and S5 Table).

The enhancement effects seen in the loss of cell viability with Cu(Picolinyl-L-Tyrosinate)_2_ were dose-dependent after irradiation at both kV and MV range. In non-irradiated control cells, the 100 μM of Cu(Picolinyl-L-Tyrosinate)_2_ decreased the cell proliferation 1.2 times (*P* < 0.01) more effectively, compared to 10 μM (Fig 7C and S6 Table).

### Assessment of the effect of Cu(Isonicotinyl-L-Tyrosinate)_2_ on cells

**Fig 8:**
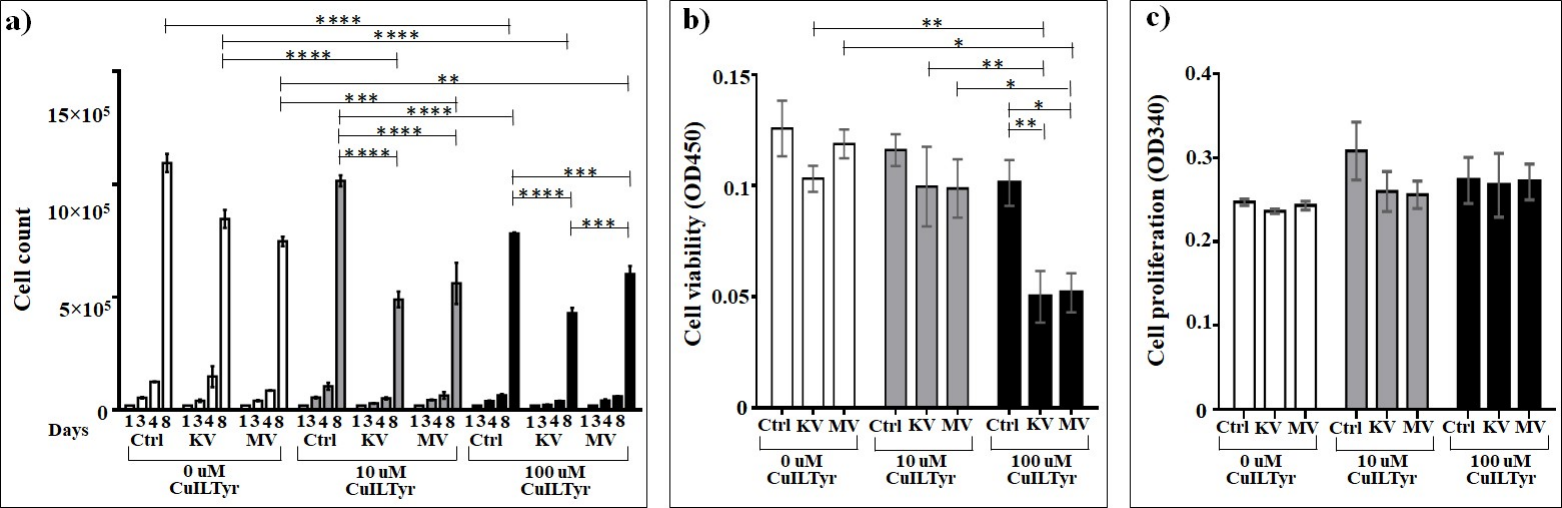
The effect of Cu(Isonicotinyl-L-Tyrosinate)_2_ on A549 lung carcinoma epithelial cells irradiated or non-irradiated with kV or MV irradiation. a) Cumulative cells growth; b) WST-1 cell viability assay; c) BrdU cell proliferation assay. *The graphs show the mean ± standard error of the mean (M ± SEM)*. *P < 0.05; **P < 0.01; ***P < 0.*001*.

The results obtained for the effect of Cu(Isonicotinyl-L-Tyrosinate)_2_ (Fig 8A and S7 Table) showed that in concentration of 100 μM it 1.4 and 1.3 times significantly decelerates the cell growth from forth to eighth days compared to non-irradiated non-treated cells and those treated with 10 μM, respectively. In case of irradiation at kV range, the treatment with 10 μM or 100 μM of this compound significantly decreased the cell growth on days four (2.84 and 3.75 times for 10 μM and 100 μM concentrations, respectively) and eight (1.73 and 2 times for 10 μM and 100 μM concentrations, respectively) compared to non-irradiated non-treated cells. Similarly, nearly the same profile was observed in case of MV irradiation, where treatment with 10 μM or 100 μM of this compound significantly decreased the cell growth only on day eight (1.33 and 1.24 times for 10 μM and 100 μM concentrations, respectively) compared to non-treated non-irradiated cells. Subsequently, the treatment with 10 μM of Cu(Isonicotinyl-L-Tyrosinate)_2_ 2.1 and 1.8 times markedly decreases the growth of the cells irradiated in kV or MV range compared to non-irradiated treated cells, respectively. Similarly, the treatment with 100 μM 1.82 and 1.3 times significantly suppressed the growth of cells irradiated at kV or MV range, respectively, compared to the non-irradiated treated cells. However, interestingly, the suppression in case of kV irradiation was 1.4 times markedly more intense compared to MV irradiation (Fig 8A and S7 Table).

Compared to irradiation alone, the addition of 10 μM Cu(Isonicotinyl-L-Tyrosinate)_2_ resulted in a 1.7 times significant (*P* < 0.0001) increase in cell death at the kV and a 1.4 times significant (*P* < 0.001) increase at the MV rage. For 100 μM, the significant enhancement in cell death was 2 times (*P* < 0.0001) at the kV and 1.3 times (*P* < 0.01) at the MV range (based on cell counts day 8).

No statistically significant changes were seen as effect of Cu(Isonicotinyl-L-Tyrosinate)_2_ on the cell viability assessed by the WST-1 test, compared to irradiation alone (*P* > 0.05) at either kV and MV ranges in case of 10 μM Cu(Isonicotinyl-L-Tyrosinate)_2_, while the addition of 100 μM Cu(Isonicotinyl-L-Tyrosinate)_2_ resulted in a 2 times significant (*P* < 0.01) decrease in cell viability at the kV range and a 2.3 times significant (*P* < 0.05) decrease at MV range. And subsequently, in both concentrations, no any differences were observed between the kV and MV ranges (Fig 8B and S8 Table).

The enhancement effects seen in the loss of cell viability with Cu(Isonicotinyl-L-Tyrosinate)_2_ were dose-dependent after irradiation at both kV and MV range. No statistically significant differences were seen between kV and MV irradiation. No statistically significant differences on proliferation as effect of Cu(Isonicotinyl-L-Tyrosinate)_2_ were observed (Fig 8C).

### Assessment of the effect of Cu(Picolinyl-L-Tryptophanate)_2_ on cells

**Fig 9:**
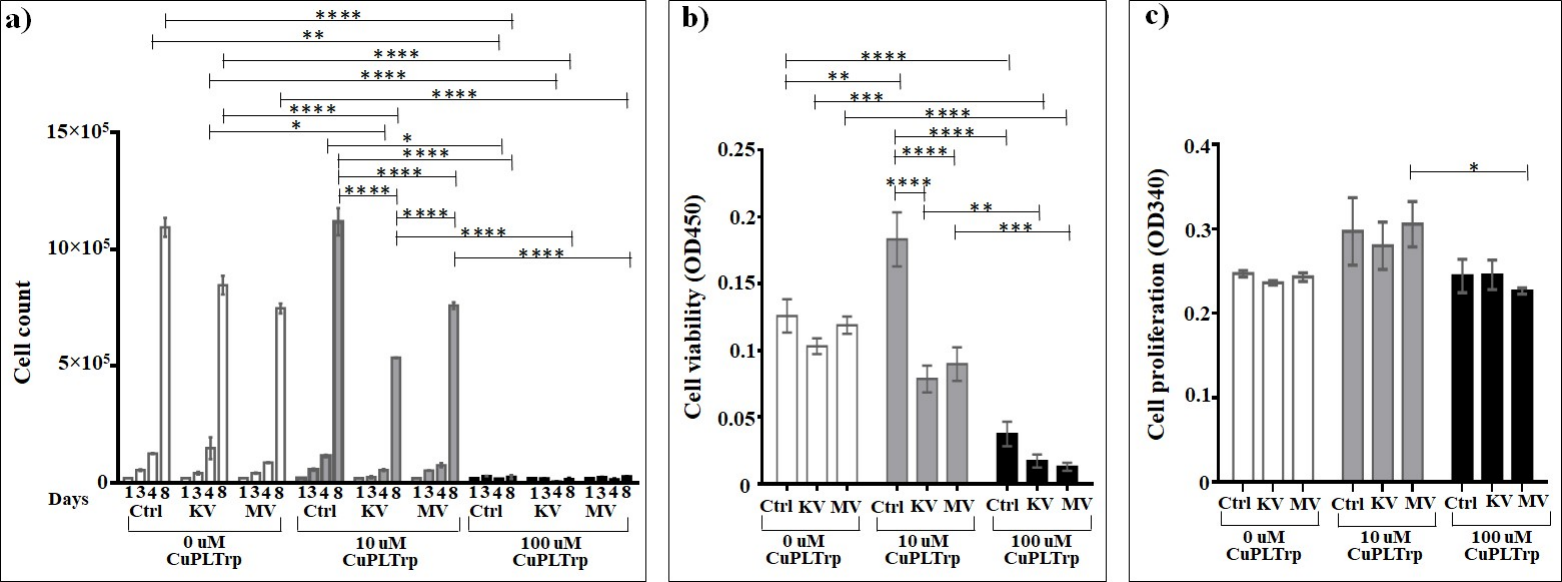
The effect of Cu(Picolinly-L-Tryptophanate)_2_ on A549 lung carcinoma epithelial cells irradiated or non-irradiated with kV or MV irradiation. a) Cumulative cells growth; b) WST-1 cell viability assay; c) BrdU cell proliferation assay. *The graphs show the mean ± standard error of the mean (M ± SEM)*. *P < 0.05; **P < 0.01; ***P < 0.001; ****P < 0.*0001*.

The assessment of the impact of the Cu(Picolinyl-L-Tryptophanate)_2_ (Fig 9A and S9 Table) demonstrated that this compound itself (without irradiation) does not affect the cell growth in concentration of 10 μM but affects in concentration of 100 μM on days four (6.76 times) and eight (43.1 times), with the subsequent difference (6.2 and 60.4 times for days four and eight, respectively) between the effects of 10 μM and 100 μM concentrations. In case of irradiation of the cells at kV range, this compound decelerates the cell growth not only in both concentrations on days four (2.7 and 24.1 times for 10 μM and 100 μM concentrations, respectively) and eight (1.6 and 51.4 times for 10 μM and 100 μM concentrations, respectively) compared to kV irradiated non-treated cells, but also demonstrates 32.5 times more intense suppression in concentration of 100 μM compared to 10 μM on day eight. Interestingly, in case of irradiation at MV range, Cu(Picolinyl-L-Tryptophanate)_2_ decreases (26.6 times) the growth of the cells only in concentration of 100 μM and only on day eight compared to MV irradiated non-treated cells, with the subsequent more intense suppression of cell growth compared to 10 μM. Although the 10 μM of Cu(Picolinyl-L-Tryptophanate)_2_ significantly decreases the cell growth under both irradiation ranges (2.1 and 1.5 times for kV and MV ranges of irradiation, respectively), compared to the non-irradiated treated cells, interestingly, it decelerates the cell growth 1.42 times significantly more intense under the irradiation at kV range than in MV range.

Compared to irradiation alone, the addition of 10 μM Cu(Picolinyl-L-Tryptophanate)_2_ resulted in a 1.6 times significant (*P* < 0.0001) increase in cell death at the kV and no statistically significant change (*P* > 0.05) at the MV rage. For 100 μM, the significant enhancement in cell death was 51 times (*P* < 0.0001) at the kV and 27 times (*P* < 0.0001) at the MV range (based on cell counts day 8).

No statistically significant changes were seen as effect of Cu(Picolinyl-L-Tryptophanate)_2_ on the cell viability assessed by the WST-1 test, compared to irradiation alone. The addition of 10 μM Cu(Picolinyl-L-Tryptophanate)_2_ resulted in a 1.3 times decrease in cell viability at both kV and MV ranges, which however was statistically not significant (*P* > 0.05). In contrast, the addition of 100 μM Cu(Picolinyl-L-Tryptophanate)_2_ resulted in a 6 times significant (*P* < 0.001) decrease in cell viability at the kV range and a 9 times significant (*P* < 0.0001) decrease at the MV range. And subsequently, in both concentrations, no any differences were observed between the kV and MV ranges (Fig 9B and S10 Table).

Interestingly, in the non-irradiated control cells the 10 μM of this compound resulted in an1.5 times increase in cell viability, which was statistically significant (*P* < 0.01), while 100 μM resulted in statistically significant (*P* < 0.01) decrease in the cell viability compared to the non-irradiated non-treated cells (Fig 9B and S10 Table).

The enhancement effects seen in the loss of cell viability with Cu(Picolinyl-L-Tryptophanate)_2_ were dose-dependent after irradiation at both kV and MV range. We found that 100 μM of Cu(Picolinyl-L-Tryptophanate)_2_ 1.4 times decreases the cell proliferation in cells irradiated at the MV range compared to 10 μM (Fig 9C and S11 Table).

### Assessment of the effect of Cu(Picolinyl-L-Phenylalaninate)_2_ on cells

**Fig 10:**
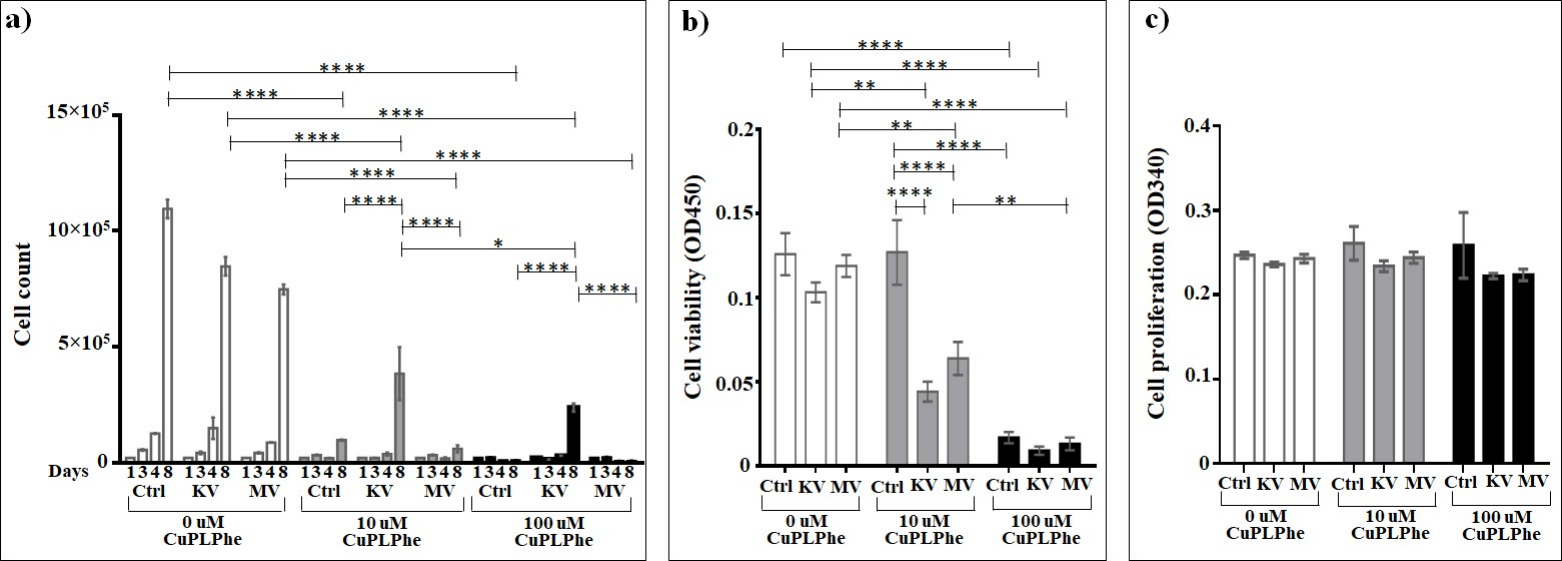
The effect of Cu(Picolinyl-L-Phenylalaninate)_2_ on A549 lung carcinoma epithelial cells irradiated or non-irradiated with kV or MV irradiation. a) Cumulative cells growth; b) WST-1 cell viability assay; c) BrdU cell proliferation assay. *The graphs show the mean ± standard error of the mean (M ± SEM)*. *P < 0.05; **P < 0.01; ***P < 0.001; ****P < 0.*0001*.

According to the results obtained for Cu(Picolinyl-L-Phenylalaninate)_2_ (Fig 10A and S12 Table), itsboth concentrations strongly suppress the cell growth in non-irradiated cells compared to non-irradiated non-treated cells (6.4 and 11.2 times for 10 μM, and 12.8 and 106 times for 100 μM on days four and eight, respectively) with 9.4 times significantly more intense suppression under the 100 μM conditions than 10 μM. Nearly the same profile is observed in case of irradiation at kV range, under which 10 μM of this compound decreases the cell growth by 4.2 and 2.2 times, and 100 μM decreases by 5 and 3.4 times on days four and eight, respectively. Moreover, the 10 μM accelerate the cell growth 1.6 times more than 100 μM. In case of MV irradiation, the same profile is observed only on day eight, when this compound decreases the cell growth by 12.6 and 104.5 times in concentrations of 10 μM and 100 μM, compared to irradiated non-treated cells. Very interesting results are obtained within the groups of different concentrations, which again have the same profile. Thus, both concentrations of this compound significantly increase (3.9 and 23 times for 10 μM and 100 μM, respectively) the growth of the cells irradiated at kV range on day eight compared to non-irradiated treated cells. When comparing cell growth between the conditions of irradiation at kV or MV ranges, the Cu(Picolinyl-L-Phenylalaninate)_2_ suppresses the cell growth more efficiently (6.5 and 33.2 times in case of 10 μM and 100 μM, respectively) under the MV irradiation than under the kV irradiation.

Compared to irradiation alone, the addition of 10 μM Cu(Picolinyl-L-Phenylalaninate)_2_ resulted in a 2.2 times significant (*P* < 0.0001) increase in cell death at the kV and a 13 times significant (*P* < 0.0001) increase at the MV rage. For 100 μM, the significant enhancement in cell death was 4 times (*P* < 0.0001) at the kV and 105 times (*P* < 0.0001) at the MV range (based on cell counts day 8).

No statistically significant changes were seen as effect of Cu(Picolinyl-L-Phenylalaninate)_2_ on the cell viability assessed by the WST-1 test, compared to irradiation alone. The addition of 10 μM Cu(Picolinyl-L-Phenylalaninate)_2_ resulted in a 2.3 times significant (*P* < 0.01) decrease in cell viability at kV range and in a 2 times significant (*P* < 0.001) decrease at MV range, while the addition of 100 μM Cu(Picolinyl-L-Phenylalaninate)_2_ resulted in a 11.4 times significant (*P* < 0.0001 decrease in cell viability at the kV range and a 9 times significant (*P* < 0.0001) decrease at the MV range. And subsequently, in both concentrations, no any differences were observed between the kV and MV ranges (Fig 10B and S13 Table).

The enhancement effects seen in the loss of cell viability with Cu(Picolinyl-L-Phenylalaninate)_2_ were dose-dependent after irradiation at both kV and MV range.

### Assessment of the effect of Cu(Nicotinyl-L-Phenylalaninate)_2_ on cells

**Fig 11:**
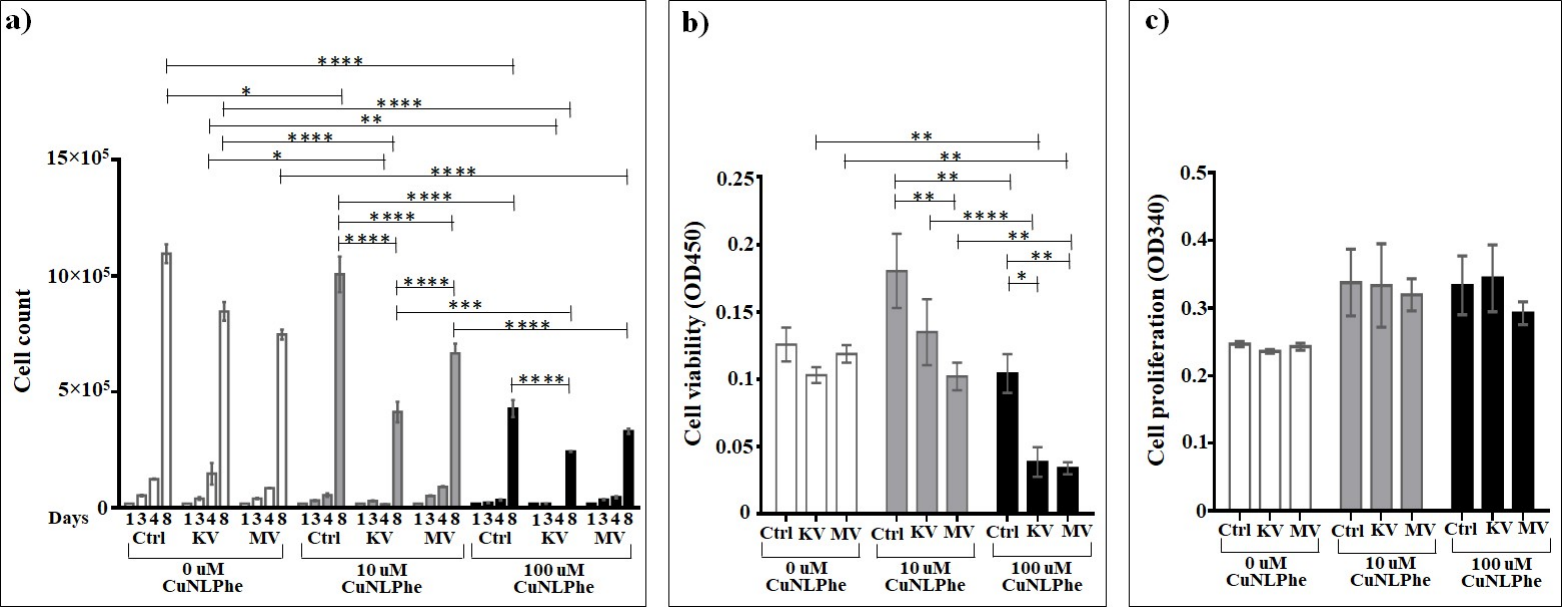
The effect of Cu(Nicotinyl-L-Phenylalaninate)_2_ on A549 lung carcinoma epithelial cells irradiated or non-irradiated with kV or MV irradiation. a) Cumulative cells growth; b) WST-1 cytotoxicity assay; c) BrdU cell proliferation assay. *The graphs show the mean ± standard error of the mean (M ± SEM)*. *P < 0.05; **P < 0.01; ***P < 0.001; ****P < 0.*0001*.

The investigation of the influence of Cu(Nicotinyl-L-Phenylalaninate)_2_ (Fig 11A and S14 Table) on the growth of non-irradiated cells demonstrated that in concentration of 10 μM this compound has a very little (1.09 times), however significant impact compared to non-irradiated non-treated cells only on day eight, in contrast to the 100 μM, which 2.4 and 2.55 times significantly decreases the cell growth on days four and eight, respectively. Subsequently, the 100 μM concentration of Cu(Nicotinyl-L-Phenylalaninate)_2_ suppresses cell growth 2.3 times significantly more intense than 10 μM. In case of irradiation at both kV and MV rages, both concentrations decrease the cell number on days four and eight. Particularly, in case of kV irradiation, 10 μM concentration decreases the cell number by 8.8 and 2 times, and 100 μM decreases by 25.3 and 3.5 times on day four and eight, respectively, compared to non-treated kV-irradiated cells, while 100 μM decreases the cell growth 1.7 times significantly more than 10 μM only on from fourth to eighth days. With regard to MV irradiation, 10 μM of Cu(Nicotinyl-L-Phenylalaninate)_2_ has a very little (1.1 times) however significant (*P* < 0.05) suppressing impact, and the 100 μM 2.3 times significantly decreases the cell growth on the cell growth on day 8 compared to non-treated kV-irradiated cells. And subsequently, the 100 μM concentration decreases the cell growth 2 times more efficiently than 10 μM. Further, compared to treated non-irradiated controls, both concentrations decrease the growth of the cells exposed to kV (2.4 and 4.1 times for 10 μM and 100 μM, respectively) or MV irradiation (1.5 and 3 times for 10 μM and 100 μM, respectively) on day eight. Interestingly, both of the concentrations more efficiently suppress the cell growth under the kV-irradiation than under MV-irradiation (1.6 and 1.4 times for 10 μM and 100 μM, respectively) on day 8.

Compared to irradiation alone, the addition of 10 μM Cu(Nicotinyl-L-Phenylalaninate)_2_ resulted in a 2 times significant (*P* < 0.0001) increase in cell death at the kV and no statistically significant change at the MV rage. For 100 μM, the significant enhancement in cell death was 3.5 times (*P* < 0.0001) at the kV and 2.3 times (*P* < 0.0001) at the MV range (based on cell counts day 8).

No statistically significant changes were seen as effect of Cu(Nicotinyl-L-Phenylalaninate)_2_ on the cell viability assessed by the WST-1 test, compared to irradiation alone. The addition of 10 μM Cu(Nicotinyl-L-Phenylalaninate)_2_ did not lead to any statistically significant (*P* > 0.05) changes in cell viability at kV and MV ranges, while the addition of 100 μM Cu(Nicotinyl-L-Phenylalaninate)_2_ resulted in a 2.6 times significant (*P* < 0.05) decrease in cell viability at the kV range and a 3.5 times significant (*P* < 0.01) decrease at the MV range. Meanwhile, in both concentrations, no any differences were observed between the kV and MV ranges (Fig 11B and S15 Table). The enhancement effects seen in the loss of cell viability with Cu(Nicotinyl-L-Phenylalaninate)_2_ were dose-dependent after irradiation at both kV and MV range.

### Assessment of the effect of Cu(Isonicotinyl-L-Phenylalaninate)_2_ on cells

**Fig 12:**
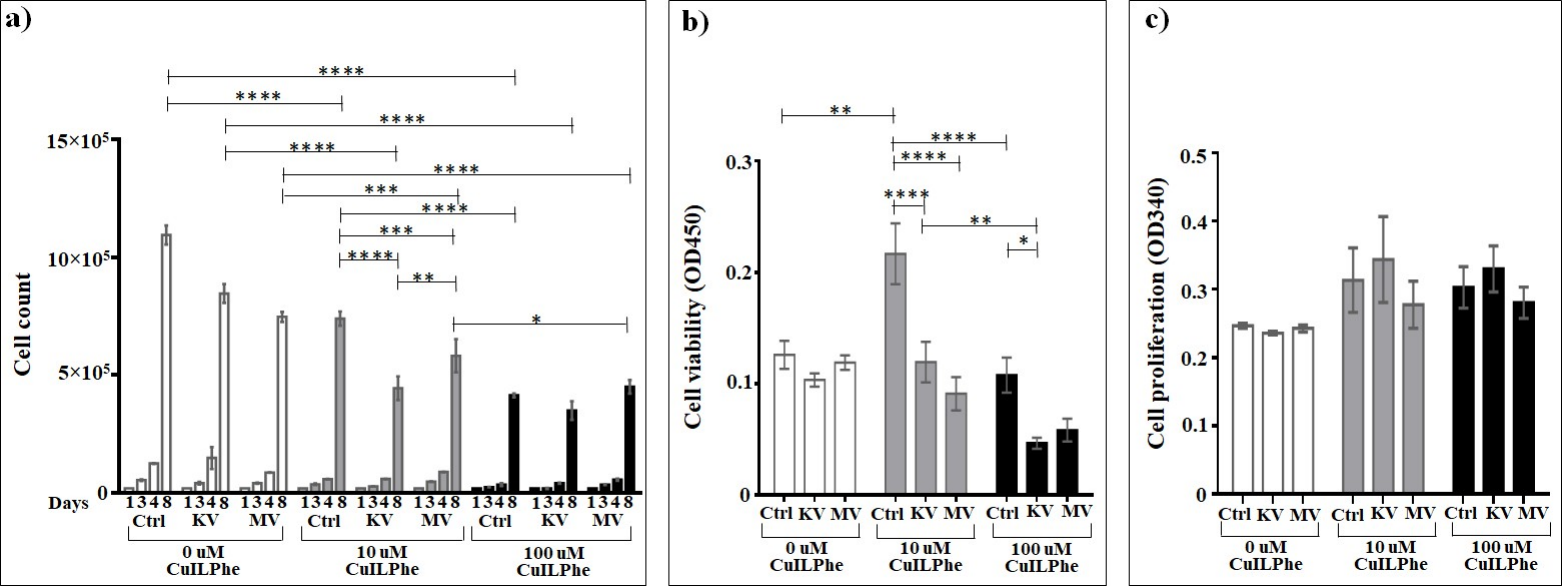
The effect of Cu(Isonicotinyl-L-Phenylalaninate)_2_ on A549 lung carcinoma epithelial cells irradiated or non-irradiated with kV or MV irradiation. a) Cumulative cells growth; b) WST-1 cell viability assay; c) BrdU cell proliferation assay. *The graphs show the mean ± standard error of the mean (M ± SEM)*. *P < 0.05; **P < 0.01; ***P < 0.001; ****P < 0.*0001*.

The investigation of the influence of Cu(Isonicotinyl-L-Phenylalaninate)_2_ (Fig 12A and S16 Table) on the growth of non-irradiated cells demonstrated that both 10 μM (1.5 times on eighth day) and 100 μM (3.5 times and 2.6 times on fourth and eighth days) concentrations of this compound affect the cell growth in non-irradiated cells compared to non-irradiated non-treated cells. In this, 100 μM affect the cell growth 1.8 times more than 10 μM. The same profile is observed when the cells are exposed to irradiation at kV or MV ranges. Thus, in case of kV irradiation, 10 μM treatment decreases the cell number by 2.5 and 1.9 times, and 100 μM decreases 3.5 and 2.4 times on days four and eight, respectively, compared to non-treated kV-irradiated cells, while in case of MV irradiation these two concentrations significantly decrease the cell growth 1.3 and 1.7 times only on the day eight, respectively. Moreover, for both types of irradiations the difference between the impact of these two concentrations on eighth day is the same, 1.3 times. However, 10 μM of Cu(Isonicotinyl-L-Phenylalaninate)_2_ 1.7 and 1.3 times significantly affects the cell growth under kV or MV irradiation on day eight, respectively, compared to treated non-irradiated cells, where the suppression of cell growth on day eight is 1.3 times more efficient in case of kV irradiation than in case of MV irradiation. In contrast, the 100 μM treatment was 1.3 times more efficient under MV irradiation than under kV-irradiation.

Compared to irradiation alone, the addition of 10 μM Cu(Isonicotinyl-L-Phenylalaninate)_2_ resulted in a 2 times significant (*P* < 0.0001) increase in cell death at the kV and a 1.3 times significant (*P* < 0.001) increase at the MV rage. For 100 μM, the significant enhancement in cell death was 2.4 times (*P* < 0.0001) at the kV and 1.7 times (*P* < 0.0001) at the MV range (based on cell counts day 8).

No statistically significant changes were seen as effect of Cu(Isonicotinyl-L-Phenylalaninate)_2_ on the cell viability assessed by the WST-1 test, compared to irradiation alone. All the differences in cell viability observed after the addition of both 10 μM and 100 μM Cu(Isonicotinyl-L-Phenylalaninate)_2_ were statistically not significant (*P* > 0.05) at both kV and MV ranges. And subsequently, in both concentrations, no any differences were observed between the kV and MV ranges (Fig 12B and S17 Table).

Interestingly and unexpectedly, the 10 μM Cu(Isonicotinyl-L-Phenylalaninate)_2_ 1.7 times significantly (*P* < 0.01) increases, while the 100 μM does not provide any significant changes (*P* > 0.05) on the cell viability compared to non-treated non-irradiated cells. However, in comparison with 10 μM, the 100 μM of this compound 2 times significantly (*P* < 0.0001) suppresses the cell viability in the non-irradiated cells. Meanwhile, the 100 μM concentration 2.6 times significantly reduce the cell viability in cells irradiated at kV range (compared to 10 μM), and significantly reduce (2 times) the cell viability in cells irradiated at MV range (compared to non-treated MV-irradiated cells). Nonetheless, in case of kV irradiation, the both treatment concentrations significantly decrease the cell viability (1.8 and 2.3 times for 10 μM and 100 μM, respectively), while in case of MV irradiation only 10 μM concentration 2.4 times suppress cell viability compared to non-irradiated treated cells (Fig 12B and S17 Table). The enhancement effects seen in the loss of cell viability with Cu(Isonicotinyl-L-Phenylalaninate)_2_ were dose-dependent after irradiation at the MV range. No significant differences were seen on proliferation as effect of Cu(Nicotinyl-L-Phenylalaninate)_2_ (Fig 12C).

## Discussion

Radiotherapy is an important treatment modality for solid tumors [39]. Clinical radiotherapy uses ionizing radiation to produce free radicals, DNA damage and finally cause cell death [40, 41]. Some cancers are, unfortunately, fairly radioresistant [42, 43]. For patients with lung cancer, average survival time is approximately 2.5 years [44]. Radioenhancers are a suitable approach to increase cancer cell destruction, to achieve higher efficiency/cytotoxicity in cancer treatment and lower damage/toxicity to healthy tissue, respectively [45]. Owing to the specific characteristics of such compounds, their efficacy might be energy-depended. Studies have compared different high- and low-energy radiation techniques, using 4 or 6 MV X-rays, ^137^Cs (^137^Cesium) or ^60^Co (^60^Cobalt) [46]. In this context it was shown that irradiation with different techniques and at different energy ranges, despite the same nominal X-ray dose being administered, provide different effects on biological samples. There is only limited literature available, comparing the effects of irradiation at the kV and MV ranges on cells or tissue [46–50]. This study provides additional information on the effectiveness of irradiation at the kV and MV ranges on cancer cells with respect to radioenhancement.

In the last few years, increasing attention has been given to the combination of radiotherapy with radioenhancers (targeting the tumor tissue) or radioprotector metal compounds (targeting the normal tissue). It has been shown that there is a dysregulation of copper homeostasis and/or localization in the organism affected by cancer [51, 52]. Therefore, copper (II) complexes have received increasing attention for their biological characteristics [10, 31, 53–57]. The development of copper-based metal complexes may promise an alternative to the adverse effects and toxicities seen in the traditionally used chemotherapy agent [10, 31, 53–57].

In this paper we have described the synthesis and structural characterization of six Schiff base copper (II) complexes, Cu(Picolinyl-L-Tryptopahanate)_2_, Cu(Picolinyl-L-Tyrosinate)_2_, Cu(Isonicotinyl-L-Tyrosinate)_2_, Cu(Picolinyl-L-Phenylalaninate)_2_, Cu (Nicotinyl-L-Phenylalaninate)_2_ and Cu(Isonicotinyl-L-Phenylalaninate)_2._ Their molecular weights were calculated, their 2D and 3D structures were predicted *in silico*. Based on those data, the designed compounds are copper (II) complexes containing two Schiff base ligands. To explore the potential radioenhancement capacity of the newly synthesized compounds as a basis for use in cancer treatment, their impact on cell growth, cell viability and cell proliferation was studied in cell cultures of human lung carcinoma cells. The copper (II) complexes themselves are not soluble in water, only in DMSO. In order to avoid the cytotoxic effect of DMSO on the cells, we decided to work with a colloidal solution in water instead. The colloidal solutions were added to the cell cultures for a period of 24 hours, after which it was removed just prior to irradiation. This was done to ensure that the enhancement effect was due to the compounds already incorporated into or attached to the tumor cells, not a secondary effect generated by free compound in solution.

The aim of these experiments was to identify possible radioenhancers for future clinical radiotherapy. The two irradiation systems utilized in our experiments represent typical equipment in clinical radiooncology. All patients with deep-seated tumors are usually irradiated at Linacs operating in the MV range. Only superficial malignant lesions are occasionally still irradiated in the kV range. Moreover, a radioenhancement effect can be energy-depend. In other words, the radioenhancement capacity of a compound may be different for irradiation at the same target dose in the kV as compared to the MV range. The data obtained showed that all the tested compounds possess radioenhancement features in different concentrations and under different irradiation conditions. All compounds exerted very potent radioenhancer capacities in the irradiated lung carcinoma cells at both the kV and MV range in a 100 μM concentration. At a concentration of 10 μM, only Cu(Picolinyl-L-Tyrosinate)_2_, Cu(Isonicotinyl-L-Tyrosinate)_2_, Cu(Picolinyl-L-Phenylalaninate)_2_ possessed radioenhancer properties at both the kV and MV ranges. Cu(Picolinyl-L-Tryptophanate)_2_ showed radioenhancer properties only at the kV range. Cu(Nicotinyl-L-Phenylalaninate)_2_ and Cu(Isonicotinyl-L-Phenylalaninate)_2_ showed remarkable radioenhancer activity only at the MV range. It should be mentioned that all compounds acted in dose-dependent manner at both tested energy ranges. Data obtained using human colon cancer cell line HT-29 from American Type Culture Collection (ATCC^®^ HTB-38™) support our conclusion regarding the radioenhancement capabilities of the compounds tested, as provided in S18–S24 Tables.

It is known that copper (II) complexes are redox active and initiate the generation of reactive oxygen species while entering the cell, thereby activating downstream mechanisms of apoptosis and cell death [10, 54]. Thus, we believe that the most important results in our study are those obtained with the viability test, because the last time point for the cell count, only five days after irradiation, might have been too early to show the full effects of irradiation-induced late cell death. The results of the proliferation test show that those cells that are still viable have almost the full proliferation potential.

1 Gy was used for irradiation studies as a standard dose in toxicity studies. Higher doses might result in too high a degree of cell destruction with little room to demonstrate further cell destruction caused by the dose enhancement. In our studies, an irradiation dose of 1 Gy affects the growth curve and cell viability at both the kV and MV ranges (Fig 6A and 6B). There appears to be no change in cell proliferation, showing that the cells surviving the irradiation do still have an almost full proliferative potential (Fig 6C). A single dose of 1 Gy is insufficient to trigger irreparable damage in all tumor cells. In a clinical setting, more than one irradiation fraction would be administered to eliminate all viable tumor cells. Given the relatively good decrease in tumor cell viability seen with some of the compounds tested, it would be worthwhile to consider a future *in vitro* study to understand whether a dose sparing effects can be achieved compared to normofractionation (2Gy / fraction).

Irradiation at both energy ranges resulted in almost the same cumulative cell numbers, with a statistically not significant tendency of higher cytotoxicity at the MV range, represented as an overall decreased cell population (Fig 6). These effects are in line with previously described findings [47, 50] showing that cancer cells are more susceptible to kV irradiation than 6 MV irradiation with the same doses. Irradiation at the kV range is characterized by higher linear energy transfer (LET) and a denser energy deposition, which in turn may lead to more severe radiation damage to the cancer cells than MV irradiation [47].

There are several benefits of using copper (II) complexes instead of their Schiff bases. Firstly, it has been shown that both cancer tissues and the process of tumor angiogenesis are characterized by elevated need of copper, subsequently localizing the majority of the organism’s copper reserves in and around the cancer tissue [53, 58]. Taking advantage of this phenomenon, it might be possible to use copper as vehicle for tumoricidal compounds to reach and accumulate in the cancer tissue. In other words, the pathological accumulation of copper compounds in the cancer tissue might be used to develop targeted delivery of tumoricidal compounds. Moreover, it has been shown that the toxicity of the copper complexes can be directed specifically toward the tumor cells [59, 60]. Modica-Napolitano and Aprille demonstrated a higher electric potential in the plasma of cancer cells compared to healthy cells, with the tendency to have negative potential inside the cell [61]. This might allow the positively charged copper (II) complexes to accumulate specifically in the cancer cells in the organism. It has been shown that copper complexes preferentially accumulate within tumor cells, which contain negatively charged mitochondria, and are rarely observed in heart and muscle, which possess higher hydrophilicity [62, 63].

One potential limitation of this study is that the effect of the copper (II) compounds on healthy cell lines is not yet known. Such a study would be one of the next steps in our research program. One approach to limit cytotoxicity to normal cells would be administration of the copper complexes by direct intratumoral injection, as proposed by other researchers [64]. In this case, acute toxicity caused by the potential radioenhancer would be limited locally.

In recent *in vivo* studies in a small animal model of whole body irradiation, we have demonstrated that subcutaneous injection of Schiff base copper (II) complexes results in the reactivation of the irradiation-suppressed antioxidant system, modulating the immune system [32]. On this basis, we have developed the working hypothesis for our current work, postulating that the newly synthesized compounds possess radioenhancer features in cancer cells while demonstrating radioprotector and immunomodulatory capacities in a host organism. While the data obtained in this first study support the assumption that the copper (II) complexes might be suitable as radioenhancer, future studies are required to test our work hypothesis. In particular, future studies should investigate the cytotoxicity of the copper (II) compounds on normal (non-cancer) cells and look into dose sparing with clinically used X-ray doses and repeated irradiation. The latter should also answer the question whether a repeated administration of the copper (II) complexes would be required.

## Conclusions

Six Schiff base-derived new copper (II) complexes, Cu(II) (Picolinyl-L-Tryptopahanate)_2_, Cu(II) (Picolinyl-L-Tyrosinate)_2_, Cu(II) (Isonicotinyl-L-Tyrosinate)_2_, Cu(II) (Picolinyl-L-Phenylalaninate)_2_, Cu(II) (Nicotinyl-L-Phenylalaninate)_2_, and Cu(II) (Isonicotinyl-L-Phenylalaninate)_2_ have been synthesized and structurally characterized. These copper (II) complexes, in combination with 1 Gy irradiation at either 120 kV or 6 MV, are more efficient at delaying cell growth of lung cancer cells and at reducing cell viability *in vitro* than the irradiation administered alone. Thus, we have demonstrated that copper (II) complexes have a good potential for radioenhancement. Since the copper complexes were tumoricidal in a dose-dependent manner, further studies are required to investigate whether they are equally cytotoxic to normal cells and to discuss to which extent cytotoxicity affecting normal cells would be acceptable in a clinical setting.

## Supporting information

S1 Table Statistical characteristics of the cell count of the naïve A549 lung carcinoma epithelial cells in growth medium vs. added PBS, which was used as carrier for the copper complexes. Ctrl–cells in growth medium, non-irradiated; Ctrl/PBS–non-irradiated cells with PBS; kV–cells in growth medium irradiated with 1 Gy at 120 kV; kV/PBS–cells with PBS irradiated with 1 Gy at 120 kV; MV–cells in growth medium irradiated with 1 Gy at 6 MV; MV/PBS—cells with PBS irradiated with 1 Gy at 6 MV; *M ± SEM–mean ± standard error of the mean*.
(DOCX)

S2 Table Statistical characteristics of the WST-1 cell viability assay of the cells with PBS. Ctrl–non-treated and non-irradiated cells in growth medium; Ctrl/PBS–non-irradiated cells with PBS; kV–cells in growth medium irradiated with 1 G at 120 kV; kV/PBS–cells with PBS irradiated with 1 Gy at 120 kV; MV/PBS—cells with PBS irradiated with 1 Gy at 6 MV; *M ± SEM–mean ± standard error of the mean*.
(DOCX)

S3 Table Statistical characteristics of the BrdU cell proliferation assay of the cells with PBS irradiated with 1 Gy at 6 MV vs- non-irradiated controls. Ctrl–non-treated and non-irradiated cells; MV—non-treated cells irradiated with 1 Gy at 6 MV; *M ± SEM–mean ± standard error of the mean*.
(DOCX)

S4 Table Statistical characteristics of the cell count of the A549 lung carcinoma epithelial cells treated with Cu(Picolinyl-L-Tyrosinate)_2_. Ctrl/PBS–non-irradiated cells with PBS; kV/PBS–cells with PBS irradiated with 1 Gy at 120 kV; MV/PBS—cells with PBS irradiated with 1 Gy at 6 MV; Ctrl/CuPLTyr-10μM—non-irradiated cells treated with 10 μM Cu(Picolinyl-L-Tyrosinate)_2_; kV/CuPLTyr-10μM—cells treated with 10 μM Cu(Picolinyl-L-Tyrosinate)_2_ and irradiated with 1 Gy at 120 kV; MV/CuPLTyr-10μM—cells treated with 10 μM Cu(Picolinyl-L-Tyrosinate)_2_ and irradiated with 1 Gy at 6 MV; Ctrl/CuPLTyr-100μM—non-irradiated cells treated with 100 μM Cu(Picolinyl-L-Tyrosinate)_2_; kV/CuPLTyr-100μM—cells treated with 100 μM Cu(Picolinyl-L-Tyrosinate)_2_ and irradiated with 1 Gy at 120 kV; MV/CuPLTyr-100μM—cells treated with 100 μM Cu(Picolinyl-L-Tyrosinate)_2_ and irradiated with 1 Gy at 6 MV; *M ± SEM–mean ± standard error of the mean*.
(DOCX)

S5 Table Statistical characteristics of the WST-1 cell viability assay of the cells treated CuPLTyr. Ctrl/PBS–non-irradiated cells with PBS; kV/PBS–cells with PBS irradiated with 1 Gy at 120 kV; MV/PBS—cells with PBS irradiated with 1 Gy at 6 MV; Ctrl/CuPLTyr-10μM—non-irradiated cells treated with 10 μM Cu(Picolinyl-L-Tyrosinate)_2_; kV/CuPLTyr-10μM—cells treated with 10 μM Cu(Picolinyl-L-Tyrosinate)_2_ and irradiated with 1 Gy at 120 kV; MV/CuPLTyr-10μM—cells treated with 10 μM Cu(Picolinyl-L-Tyrosinate)_2_ and irradiated with 1 Gy at 6 MV; Ctrl/CuPLTyr-100μM—non-irradiated cells treated with 100 μM Cu(Picolinyl-L-Tyrosinate)_2_; kV/CuPLTyr-100μM—cells treated with 100 μM Cu(Picolinyl-L-Tyrosinate)_2_ and irradiated with 1 Gy at 120 kV; MV/CuPLTyr-100μM—cells treated with 100 μM Cu(Picolinyl-L-Tyrosinate)_2_ and irradiated with 1 Gy at 6 MV; *M ± SEM–mean ± standard error of the mean*.
(DOCX)

S6 Table Statistical characteristics of the BrdU cell proliferation assay of the cells treated with CuPLTyr. Ctrl/CuPLTyr-10μM—non-irradiated cells treated with 10 μM Cu(Picolinyl-L-Tyrosinate)_2_; Ctrl/CuPLTyr-100μM—non-irradiated cells treated with 100 μM Cu(Picolinyl-L-Tyrosinate)_2_; *M ± SEM–mean ± standard error of the mean*.
(DOCX)

S7 Table Statistical characteristics of the cell count of the A549 lung carcinoma epithelial cells treated with Cu(Isonicotinyl-L-Tyrosinate)_2_. Ctrl/PBS–non-irradiated cells with PBS; kV/PBS–cells with PBS irradiated with 1 Gy at 120 kV; MV/PBS—cells with PBS irradiated with 1 Gy at 6 MV; Ctrl/CuILTyr-10μM—non-irradiated cells treated with 10 μM Cu(Isonicotinyl-L-Tyrosinate)_2_; kV/CuILTyr-10μM—cells treated with 10 μM Cu(Isonicotinyl-L-Tyrosinate)_2_ and irradiated with 1 Gy at 120 kV; MV/CuILTyr-10μM—cells treated with 10 μM Cu(Isonicotinyl-L-Tyrosinate)_2_ and irradiated with 1 Gy at 6 MV; Ctrl/CuILTyr-100μM—non-irradiated cells treated with 100 μM Cu(Isonicotinyl-L-Tyrosinate)_2_; kV/CuILTyr-100μM—cells treated with 100 μM Cu(Isonicotinyl-L-Tyrosinate)_2_ and irradiated with 1 Gy at 120 kV; MV/CuILTyr-100μM—cells treated with 100 μM Cu(Isonicotinyl-L-Tyrosinate)_2_ and irradiated with 1 Gy at 6 MV; *M ± SEM–mean ± standard error of the mean*.
(DOCX)

S8 Table Statistical characteristics of the WST-1 cell viability assay of the cells treated with CuILTyr. kV/PBS–cells with PBS irradiated with 1 Gy at 120 kV; MV/PBS—cells with PBS irradiated with 1 Gy at 6 MV; kV/CuILTyr-10μM—cells treated with 10 μM Cu(Isonicotinyl-L-Tyrosinate)_2_ and irradiated with 1 Gy at 120 kV; MV/CuILTyr-10μM—cells treated with 10 μM Cu(Isonicotinyl-L-Tyrosinate)_2_ and irradiated with 1 Gy at 6 MV; Ctrl/CuILTyr-100μM—non-irradiated cells treated with 100 μM Cu(Isonicotinyl-L-Tyrosinate)_2_; kV/CuILTyr-100μM—cells treated with 100 μM Cu(Isonicotinyl-L-Tyrosinate)_2_ and irradiated with 1 Gy at 120 kV; MV/CuILTyr-100μM—cells treated with 100 μM Cu(Isonicotinyl-L-Tyrosinate)_2_ and irradiated with 1Gy at 6 MV; *M ± SEM–mean ± standard error of the mean*.
(DOCX)

S9 Table Statistical characteristics of the cell count of the A549 lung carcinoma epithelial cells treated with Cu(Picolinyl-L-Tryptophanate)_2_. Ctrl/PBS–non-irradiated cells with PBS; kV/PBS–cells with PBS irradiated with 1 Gy at 120 kV; MV/PBS—cells with PBS irradiated with 1 Gy at 6 MV; Ctrl/CuPLTrp-10μM—non-irradiated cells treated with 10 μM Cu(Picolinyl-L-Tryptophanate)_2_; kV/CuPLTrp-10μM—cells treated with 10 μM Cu(Picolinyl-L-Tryptophanate)_2_ and irradiated with 1 Gy at 120 kV; MV/CuPLTrp-10μM—cells treated with 10 μM Cu(Picolinyl-L-Tryptophanate)_2_ and irradiated with 1 Gy at 6 MV; Ctrl/CuPLTrp-100μM—non-irradiated cells treated with 100 μM Cu(Picolinyl-L-Tryptophanate)_2_; kV/CuPLTrp-100μM—cells treated with 100 μM Cu(Picolinyl-L-Tryptophanate)_2_ and irradiated with 1 Gy at 120 kV; MV/CuPLTrp-100μM—cells treated with 100 μM Cu(Picolinyl-L-Tryptophanate)_2_ and irradiated with 1 Gy at 6 MV; *M ± SEM–mean ± standard error of the mean*.
(DOCX)

S10 Table Statistical characteristics of the WST-1 cell viability assay of the cells treated with CuPLTrp with PBS and irradiated with 1 Gy at either 120 kV or 6 MV vs. non-irradiated controls. Ctrl/PBS–non-irradiated cells with PBS; kV/PBS–cells with PBS irradiated with 1 Gy at 120 kV; MV/PBS—cells with PBS irradiated with 1 Gy at 6 MV; Ctrl/CuPLTrp-10μM—non-irradiated cells treated with 10 μM Cu(Picolinyl-L-Tryptophanate)_2_; kV/CuPLTrp-10μM—cells treated with 10 μM Cu(Picolinyl-L-Tryptophanate)_2_ and irradiated with 1 Gy at 120 kV; MV/CuPLTrp-10μM—cells treated with 10 μM Cu(Picolinyl-L-Tryptophanate)_2_ and irradiated with 1 Gy at 6 MV; Ctrl/CuPLTrp-100μM—non-irradiated cells treated with 100 μM Cu(Picolinyl-L-Tryptophanate)_2_; kV/CuPLTrp-100μM—cells treated with 100 μM Cu(Picolinyl-L-Tryptophanate)_2_ and irradiated with 1 Gy at 120 kV; MV/CuPLTrp-100μM—cells treated with 100 μM Cu(Picolinyl-L-Tryptophanate)_2_ and irradiated with 1 Gy at 6 MV; *M ± SEM–mean ± standard error of the mean*.
(DOCX)

S11 Table Statistical characteristics of the BrdU cell proliferation assay of the cells treated with CuPLTrp with PBS and irradiated with 1 Gy at 6 MV vs. non-irradiated controls. MV/CuPLTrp-10μM—cells treated with 10 μM Cu(Picolinyl-L-Tryptophanate)_2_ and irradiated with 1 Gy at 6 MV; MV/CuPLTrp-100μM—cells treated with 100 μM Cu(Picolinyl-L-Tryptophanate)_2_ and irradiated with 1 Gy at 6 MV; *M ± SEM–mean ± standard error of the mean*.
(DOCX)

S12 Table Statistical characteristics of the cell count of the A549 lung carcinoma epithelial cells treated with Cu(Picolinyl-L-Phenylalaninate)_2_. Ctrl/PBS–non-irradiated cells with PBS; kV/PBS–cells with PBS irradiated with 1 Gy at 120 kV; MV/PBS—cells with PBS irradiated with 1 Gy at 6 MV; Ctrl/CuPLPhe-10μM—non-irradiated cells treated with 10 μM Cu(Picolinyl-L- Phenylalaninate)_2_; kV/CuPLPhe-10μM—cells treated with 10 μM Cu(Picolinyl-L- Phenylalaninate)_2_ and irradiated with 1 Gy at 120 kV; MV/CuPLPhe-10μM—cells treated with 10 μM Cu(Picolinyl-L- Phenylalaninate)_2_ and irradiated with 1 Gy at 6 MV; Ctrl/CuPLPhe-100μM—non-irradiated cells treated with 100 μM Cu(Picolinyl-L- Phenylalaninate)_2_; kV/CuPLPhe-100μM—cells treated with 100 μM Cu(Picolinyl-L- Phenylalaninate)_2_ and irradiated with 1 Gy at 120 kV; MV/CuPLPhe-100μM—cells treated with 100 μM Cu(Picolinyl-L-Phenylalaninate)_2_ and irradiated with 1 Gy at 6 MV; *M ± SEM–mean ± standard error of the mean*.
(DOCX)

S13 Table Statistical characteristics of the WST-1 cell viability assay of the cells exposed to CuPLPhe with PBS and irradiated with 1 Gy at either 120 kV or 6 MV vs. non-irradiated controls. Ctrl/PBS–non-irradiated cells with PBS; kV/PBS–cells with PBS irradiated with 1 Gy at 120 kV; MV/PBS—cells with PBS irradiated with 1 Gy at 6 MV; Ctrl/CuPLPhe-10μM—non-irradiated cells treated with 10 μM Cu(Picolinyl-L- Phenylalaninate)_2_; kV/CuPLPhe-10μM—cells treated with 10 μM Cu(Picolinyl-L- Phenylalaninate)_2_ and irradiated with 1 Gy at 120 kV; MV/CuPLPhe-10μM—cells treated with 10 μM Cu(Picolinyl-L- Phenylalaninate)_2_ and irradiated with 1 Gy at 6 MV; Ctrl/CuPLPhe-100μM—non-irradiated cells treated with 100 μM Cu(Picolinyl-L- Phenylalaninate)_2_; kV/CuPLPhe-100μM—cells treated with 100 μM Cu(Picolinyl-L- Phenylalaninate)_2_ and irradiated with 1 Gy at 120 kV; MV/CuPLPhe-100μM—cells treated with 100 μM Cu(Picolinyl-L-Phenylalaninate)_2_ and irradiated with 1 Gy at 6 MV; *M ± SEM–mean ± standard error of the mean*.
(DOCX)

S14 Table Statistical characteristics of the cell count of the A549 lung carcinoma epithelial cells treated with Cu(Nicotinyl-L-Phenylalaninate)_2_. Ctrl/PBS–non-irradiated cells with PBS; kV/PBS–cells with PBS irradiated with 1 Gy at 120 kV; MV/PBS—cells with PBS irradiated with 1 Gy at 6 MV; Ctrl/CuNLPhe-10μM—non-irradiated cells treated with 10 μM Cu(Nicotinyl-L- Phenylalaninate)_2_; kV/CuNLPhe-10μM—cells treated with 10 μM Cu(Nicotinyl-L- Phenylalaninate)_2_ and irradiated with 1Gy at 120 kV; MV/CuNLPhe-10μM—cells treated with 10 μM Cu(Nicotinyl-L- Phenylalaninate)_2_ and irradiated with 1Gy at 6 MV; Ctrl/CuNLPhe-100μM—non-irradiated cells treated with 100 μM Cu(Nicotinyl-L- Phenylalaninate)_2_; kV/CuNLPhe-100μM—cells treated with 100 μM Cu(Nicotinyl-L- Phenylalaninate)_2_ and irradiated with 1 Gy at 120 kV; MV/CuNLPhe-100μM—cells treated with 100 μM Cu(Nicotinyl-L-Phenylalaninate)_2_ and irradiated with 1 Gy at 6 MV; *M ± SEM–mean ± standard error of the mean*.
(DOCX)

S15 Table Statistical characteristics of the WST-1 cell viability assay of the cells exposed to CuNLPhe with PBS and irradiated with 1 Gy at either 120 kV or 6 MV vs. non-irradiated controls. kV/PBS–cells only with PBS irradiated with 1 Gy at 120 kV; MV/PBS—cells with PBS irradiated with 1 Gy at 6 MV; Ctrl/CuNLPhe-10μM—non-irradiated cells treated with 10 μM Cu(Nicotinyl-L- Phenylalaninate)_2_; kV/CuNLPhe-10μM—cells treated with 10 μM Cu(Nicotinyl-L- Phenylalaninate)_2_ and irradiated with 1Gy at 120 kV; MV/CuNLPhe-10μM—cells treated with 10 μM Cu(Nicotinyl-L- Phenylalaninate)_2_ and irradiated with 1Gy at 6 MV; Ctrl/CuNLPhe-100μM—non-irradiated cells treated with 100 μM Cu(Nicotinyl-L- Phenylalaninate)_2_; kV/CuNLPhe-100μM—cells treated with 100 μM Cu(Nicotinyl-L- Phenylalaninate)_2_ and irradiated with 1 Gy at 120 kV; MV/CuNLPhe-100μM—cells treated with 100 μM Cu(Nicotinyl-L-Phenylalaninate)_2_ and irradiated with 1 Gy at 6 MV; *M ± SEM–mean ± standard error of the mean*.
(DOCX)

S16 Table Statistical characteristics of the cell count of the A549 lung carcinoma epithelial cells treated with Cu(Isonicotinyl-L-Phenylalaninate)_2_. Ctrl/PBS–non-irradiated cells only with PBS; kV/PBS–cells with PBS irradiated with 1 Gy at 120 kV; MV/PBS–cells with PBS irradiated with 1 Gy at 6 MV; Ctrl/CuILPhe-10μM—non-irradiated cells treated with 10 μM Cu(Isonicotinyl-L- Phenylalaninate)_2_; kV/CuILPhe-10μM—cells treated with 10 μM Cu(Isonicotinyl-L- Phenylalaninate)_2_ and irradiated with 1 Gy at 120 kV; MV/CuILPhe-10μM—cells treated with 10 μM Cu(Isonicotinyl-L- Phenylalaninate)_2_ and irradiated with 1 Gy at 6 MV; Ctrl/CuILPhe-100μM—non-irradiated cells treated with 100 μM Cu(Isonicotinyl-L- Phenylalaninate)_2_; kV/CuILPhe-100μM—cells treated with 100 μM Cu(Isonicotinyl-L- Phenylalaninate)_2_ and irradiated with 1 Gy at 120 kV; MV/CuILPhe-100μM—cells treated with 100 μM Cu(Isonicotinyl-L-Phenylalaninate)_2_ and irradiated with 1 Gy at 10 MV; *M ± SEM–mean ± standard error of the mean*.
(DOCX)

S17 Table Statistical characteristics of the WST-1 cell viability assay of the cells exposed to CuILPhe with PBS and irradiated with 1 Gy at either 120 kV or 6 MV vs. non-irradiated controls. Ctrl/PBS–non-irradiated cells only with PBS; MV/PBS–cells with PBS irradiated with 1 Gy at 6 MV; Ctrl/CuILPhe-10μM—non-irradiated cells treated with 10 μM Cu(Isonicotinyl-L- Phenylalaninate)_2_; kV/CuILPhe-10μM—cells treated with 10 μM Cu(Isonicotinyl-L- Phenylalaninate)_2_ and irradiated with 1 Gy at 120 kV; MV/CuILPhe-10μM—cells treated with 10 μM Cu(Isonicotinyl-L- Phenylalaninate)_2_ and irradiated with 1 Gy at 6 MV; Ctrl/CuILPhe-100μM—non-irradiated cells treated with 100 μM Cu(Isonicotinyl-L- Phenylalaninate)_2_; kV/CuILPhe-100μM—cells treated with 100 μM Cu(Isonicotinyl-L- Phenylalaninate)_2_ and irradiated with 1 Gy at 120 kV; MV/CuILPhe-100μM—cells treated with 100 μM Cu(Isonicotinyl-L-Phenylalaninate)_2_ and irradiated with 1 Gy at 10 MV; *M ± SEM–mean ± standard error of the mean*.
(DOCX)

S18 Table Statistical characteristics of the cell count of the naïve HT-29 human colon cancer cells in growth medium vs. added PBS, which was used as carrier for the copper complexes. Ctrl–cells in growth medium, non-irradiated; Ctrl/PBS–non-irradiated cells with PBS; kV–cells in growth medium irradiated with 1 Gy at 120 kV; kV/PBS–cells with PBS irradiated with 1 Gy at 120 kV; MV–cells in growth medium irradiated with 1 Gy at 6 MV; MV/PBS—cells with PBS irradiated with 1 Gy at 6 MV; *M ± SEM–mean ± standard error of the mean*.
(DOCX)

S19 Table Statistical characteristics of the cell count of the HT-29 human colon cancer cells treated with Cu(Picolinyl-L-Tyrosinate)_2_. Ctrl/PBS–non-irradiated cells with PBS; kV/PBS–cells with PBS irradiated with 1 Gy at 120 kV; MV/PBS—cells with PBS irradiated with 1 Gy at 6 MV; Ctrl/CuPLTyr-10μM—non-irradiated cells treated with 10 μM Cu(Picolinyl-L-Tyrosinate)_2_; kV/CuPLTyr-10μM—cells treated with 10 μM Cu(Picolinyl-L-Tyrosinate)_2_ and irradiated with 1 Gy at 120 kV; MV/CuPLTyr-10μM—cells treated with 10 μM Cu(Picolinyl-L-Tyrosinate)_2_ and irradiated with 1 Gy at 6 MV; Ctrl/CuPLTyr-100μM—non-irradiated cells treated with 100 μM Cu(Picolinyl-L-Tyrosinate)_2_; kV/CuPLTyr-100μM—cells treated with 100 μM Cu(Picolinyl-L-Tyrosinate)_2_ and irradiated with 1 Gy at 120 kV; MV/CuPLTyr-100μM—cells treated with 100 μM Cu(Picolinyl-L-Tyrosinate)_2_ and irradiated with 1 Gy at 6 MV; *M ± SEM–mean ± standard error of the mean*.
(DOCX)

S20 Table Statistical characteristics of the cell count of the HT-29 human colon cancer cells treated with Cu(Isonicotinyl-L-Tyrosinate)_2_. Ctrl/PBS–non-irradiated cells with PBS; kV/PBS–cells with PBS irradiated with 1 Gy at 120 kV; MV/PBS—cells with PBS irradiated with 1 Gy at 6 MV; Ctrl/CuILTyr-10μM—non-irradiated cells treated with 10 μM Cu(Isonicotinyl-L-Tyrosinate)_2_; kV/CuILTyr-10μM—cells treated with 10 μM Cu(Isonicotinyl-L-Tyrosinate)_2_ and irradiated with 1 Gy at 120 kV; MV/CuILTyr-10μM—cells treated with 10 μM Cu(Isonicotinyl-L-Tyrosinate)_2_ and irradiated with 1 Gy at 6 MV; Ctrl/CuILTyr-100μM—non-irradiated cells treated with 100 μM Cu(Isonicotinyl-L-Tyrosinate)_2_; kV/CuILTyr-100μM—cells treated with 100 μM Cu(Isonicotinyl-L-Tyrosinate)_2_ and irradiated with 1 Gy at 120 kV; MV/CuILTyr-100μM—cells treated with 100 μM Cu(Isonicotinyl-L-Tyrosinate)_2_ and irradiated with 1 Gy at 6 MV; *M ± SEM–mean ± standard error of the mean*.
(DOCX)

S21 Table Statistical characteristics of the cell count of the HT-29 human colon cancer cells treated with Cu(Picolinyl-L-Tryptophanate)_2_. Ctrl/PBS–non-irradiated cells with PBS; kV/PBS–cells with PBS irradiated with 1 Gy at 120 kV; MV/PBS—cells with PBS irradiated with 1 Gy at 6 MV; Ctrl/CuPLTrp-10μM—non-irradiated cells treated with 10 μM Cu(Picolinyl-L-Tryptophanate)_2_; kV/CuPLTrp-10μM—cells treated with 10 μM Cu(Picolinyl-L-Tryptophanate)_2_ and irradiated with 1 Gy at 120 kV; MV/CuPLTrp-10μM—cells treated with 10 μM Cu(Picolinyl-L-Tryptophanate)_2_ and irradiated with 1 Gy at 6 MV; Ctrl/CuPLTrp-100μM—non-irradiated cells treated with 100 μM Cu(Picolinyl-L-Tryptophanate)_2_; kV/CuPLTrp-100μM—cells treated with 100 μM Cu(Picolinyl-L-Tryptophanate)_2_ and irradiated with 1 Gy at 120 kV; MV/CuPLTrp-100μM—cells treated with 100 μM Cu(Picolinyl-L-Tryptophanate)_2_ and irradiated with 1 Gy at 6 MV; *M ± SEM–mean ± standard error of the mean*.
(DOCX)

S22 Table Statistical characteristics of the cell count of the HT-29 human colon cancer cells treated with Cu(Picolinyl-L-Phenylalaninate)_2_. Ctrl/PBS–non-irradiated cells with PBS; kV/PBS–cells with PBS irradiated with 1 Gy at 120 kV; MV/PBS—cells with PBS irradiated with 1 Gy at 6 MV; Ctrl/CuPLPhe-10μM—non-irradiated cells treated with 10 μM Cu(Picolinyl-L- Phenylalaninate)_2_; kV/CuPLPhe-10μM—cells treated with 10 μM Cu(Picolinyl-L- Phenylalaninate)_2_ and irradiated with 1 Gy at 120 kV; MV/CuPLPhe-10μM—cells treated with 10 μM Cu(Picolinyl-L- Phenylalaninate)_2_ and irradiated with 1 Gy at 6 MV; Ctrl/CuPLPhe-100μM—non-irradiated cells treated with 100 μM Cu(Picolinyl-L- Phenylalaninate)_2_; kV/CuPLPhe-100μM—cells treated with 100 μM Cu(Picolinyl-L- Phenylalaninate)_2_ and irradiated with 1 Gy at 120 kV; MV/CuPLPhe-100μM—cells treated with 100 μM Cu(Picolinyl-L-Phenylalaninate)_2_ and irradiated with 1 Gy at 6 MV; *M ± SEM–mean ± standard error of the mean*.
(DOCX)

S23 Table Statistical characteristics of the cell count of the HT-29 human colon cancer cells treated with Cu(Nicotinyl-L-Phenylalaninate)_2_. Ctrl/PBS–non-irradiated cells with PBS; MV/PBS—cells with PBS irradiated with 1 Gy at 6 MV; Ctrl/CuNLPhe-10μM—non-irradiated cells treated with 10 μM Cu(Nicotinyl-L- Phenylalaninate)_2_; kV/CuNLPhe-10μM—cells treated with 10 μM Cu(Nicotinyl-L- Phenylalaninate)_2_ and irradiated with 1Gy at 120 kV; MV/CuNLPhe-10μM—cells treated with 10 μM Cu(Nicotinyl-L- Phenylalaninate)_2_ and irradiated with 1Gy at 6 MV; Ctrl/CuNLPhe-100μM—non-irradiated cells treated with 100 μM Cu(Nicotinyl-L- Phenylalaninate)_2_; MV/CuNLPhe-100μM—cells treated with 100 μM Cu(Nicotinyl-L-Phenylalaninate)_2_ and irradiated with 1 Gy at 6 MV; *M ± SEM–mean ± standard error of the mean*.
(DOCX)

S24 Table Statistical characteristics of the cell count of the HT-29 human colon cancer cells treated with Cu(Isonicotinyl-L-Phenylalaninate)_2_. Ctrl/PBS–non-irradiated cells only with PBS; kV/PBS–cells with PBS irradiated with 1 Gy at 120 kV; MV/PBS–cells with PBS irradiated with 1 Gy at 6 MV; Ctrl/CuILPhe-10μM—non-irradiated cells treated with 10 μM Cu(Isonicotinyl-L- Phenylalaninate)_2_; kV/CuILPhe-10μM—cells treated with 10 μM Cu(Isonicotinyl-L- Phenylalaninate)_2_ and irradiated with 1 Gy at 120 kV; MV/CuILPhe-10μM—cells treated with 10 μM Cu(Isonicotinyl-L- Phenylalaninate)_2_ and irradiated with 1 Gy at 6 MV; Ctrl/CuILPhe-100μM—non-irradiated cells treated with 100 μM Cu(Isonicotinyl-L- Phenylalaninate)_2_; kV/CuILPhe-100μM—cells treated with 100 μM Cu(Isonicotinyl-L- Phenylalaninate)_2_ and irradiated with 1 Gy at 120 kV; MV/CuILPhe-100μM—cells treated with 100 μM Cu(Isonicotinyl-L-Phenylalaninate)_2_ and irradiated with 1 Gy at 10 MV; *M ± SEM–mean ± standard error of the mean*.
(DOCX)
