# Supplementary material for: The radioenhancement potential of Schiff base derived copper (II) compounds against lung carcinoma in vitro
Source: PLoS One. 2021 Jun 18;16(6):e0253553. doi: 10.1371/journal.pone.0253553 (PMC8213134; doi:10.1371/journal.pone.0253553)
Supplement: S1 Table — Ctrl–cells in growth medium, non-irradiated; Ctrl/PBS–non-irradiated cells with PBS; kV–cells in growth medium irradiated with 1 Gy at 120 kV; kV/PBS–cells with PBS irradiated with 1 Gy at 120 kV; MV–cells in growth medium irradiated with 1 Gy at 6 MV; MV/PBS—cells with PBS irradiated with 1 Gy at 6 MV; M ± SEM–mean ± standard error of the mean. (DOCX) [file pone.0253553.s001.docx]

**S1 Table**. **Statistical characteristics of the cell count of the naïve A549 lung carcinoma epithelial cells in growth medium vs. added PBS, which was used as carrier for the copper complexes.** Ctrl – cells in growth medium, non-irradiated; Ctrl/PBS – non-irradiated cells with PBS; kV – cells in growth medium irradiated with 1 Gy at 120 kV; kV/PBS – cells with PBS irradiated with 1 Gy at 120 kV; MV – cells in growth medium irradiated with 1 Gy at 6 MV; MV/PBS - cells with PBS irradiated with 1 Gy at 6 MV; *M ± SEM – mean ± standard error of the mean.*

| **Group** | **Days** | **М±SEM** | **Compared groups** | **Difference (times)** | ***P*** |
| --- | --- | --- | --- | --- | --- |
| **Ctrl** | **Day 8** | 1100750 ± 48250 | Ctrl vs. kV | 1.3 | < 0.0001 |
|  |  |  | Ctrl vs. MV | 1.4 | <0.0001 |
| **Ctrl/PBS** | **Day 8** | 1094750 ± 40250 | Ctrl/PBS vs. kV/PBS | 1.3 | <0.0001 |
|  |  |  | Ctrl/PBS vs. MV/PBS | 1.5 | <0.0001 |
| **kV** | **Day 8** | 862000 ± 26500 |  |  |  |
| **kV/PBS** | **Day 8** | 846750 ± 40250 |  |  |  |
| **MV** | **Day 8** | 777750 ± 84750 |  |  |  |
| **MV/PBS** | **Day 8** | 747250 ± 20750 |  |  |  |
| **Ctrl/PBS** | **Day 4** | 125200 ± 1700 |  |  |  |
| **kV/PBS** | **Day 4** | 148700 ± 46400 |  |  |  |
