# Supplementary material for: The radioenhancement potential of Schiff base derived copper (II) compounds against lung carcinoma in vitro
Source: PLoS One. 2021 Jun 18;16(6):e0253553. doi: 10.1371/journal.pone.0253553 (PMC8213134; doi:10.1371/journal.pone.0253553)
Supplement: S2 Table — Ctrl–non-treated and non-irradiated cells in growth medium; Ctrl/PBS–non-irradiated cells with PBS; kV–cells in growth medium irradiated with 1 G at 120 kV; kV/PBS–cells with PBS irradiated with 1 Gy at 120 kV; MV/PBS—cells with PBS irradiated with 1 Gy at 6 MV; M ± SEM–mean ± standard error of the mean. (DOCX) [file pone.0253553.s002.docx]

**S2 Table. Statistical characteristics of the WST-1 cell viability assay of the cells with PBS.** Ctrl – non-treated and non-irradiated cells in growth medium; Ctrl/PBS – non-irradiated cells with PBS; kV – cells in growth medium irradiated with 1 G at 120 kV; kV/PBS – cells with PBS irradiated with 1 Gy at 120 kV; MV/PBS - cells with PBS irradiated with 1 Gy at 6 MV; *M ± SEM – mean ± standard error of the mean.*

| **Group** | **М±SEM** | **Compared groups** | **Difference (times)** | ***P*** |
| --- | --- | --- | --- | --- |
| **Ctrl** | 0.136 ± 0.010 | Ctrl vs. kV | 1.62 | < 0.05 |
| **Ctrl/PBS** | 0.126 ± 0.013 |  |  |  |
| **kV** | 0.084 ± 0.008 |  |  |  |
| **kV/PBS** | 0.103 ± 0.006 |  |  |  |
| **MV/PBS** | 0.119 ± 0.007 |  |  |  |
