# Supplementary material for: The radioenhancement potential of Schiff base derived copper (II) compounds against lung carcinoma in vitro
Source: PLoS One. 2021 Jun 18;16(6):e0253553. doi: 10.1371/journal.pone.0253553 (PMC8213134; doi:10.1371/journal.pone.0253553)
Supplement: S3 Table — Ctrl–non-treated and non-irradiated cells; MV—non-treated cells irradiated with 1 Gy at 6 MV; M ± SEM–mean ± standard error of the mean. (DOCX) [file pone.0253553.s003.docx]

**S3 Table. Statistical characteristics of the BrdU cell proliferation assay of the cells with PBS irradiated with 1 Gy at 6 MV vs- non-irradiated controls.** Ctrl – non-treated and non-irradiated cells; MV - non-treated cells irradiated with 1 Gy at 6 MV; *M ± SEM – mean ± standard error of the mean.*

| **Group** | **М±SEM** | **Compared groups** | **Difference (times)** | ***P*** |
| --- | --- | --- | --- | --- |
| **Ctrl** | 0.231 ± 0.003 | Ctrl vs. MV | 1.1 | < 0.05 |
| **MV** | 0.250 ± 0.007 |  |  |  |
