# Supplementary material for: The radioenhancement potential of Schiff base derived copper (II) compounds against lung carcinoma in vitro
Source: PLoS One. 2021 Jun 18;16(6):e0253553. doi: 10.1371/journal.pone.0253553 (PMC8213134; doi:10.1371/journal.pone.0253553)
Supplement: S4 Table — Ctrl/PBS–non-irradiated cells with PBS; kV/PBS–cells with PBS irradiated with 1 Gy at 120 kV; MV/PBS—cells with PBS irradiated with 1 Gy at 6 MV; Ctrl/CuPLTyr-10μM—non-irradiated cells treated with 10 μM Cu(Picolinyl-L-Tyrosinate)2; kV/CuPLTyr-10μM—cells treated with 10 μM Cu(Picolinyl-L-Tyrosinate)2 and irradiated with 1 Gy at 120 kV; MV/CuPLTyr-10μM—cells treated with 10 μM Cu(Picolinyl-L-Tyrosinate)2 and irradiated with 1 Gy at 6 MV; Ctrl/CuPLTyr-100μM—non-irradiated cells treated with 100 μM Cu(Picolinyl-L-Tyrosinate)2; kV/CuPLTyr-100μM—cells treated with 100 μM Cu(Picolinyl-L-Tyrosinate)2 and irradiated with 1 Gy at 120 kV; MV/CuPLTyr-100μM—cells treated with 100 μM Cu(Picolinyl-L-Tyrosinate)2 and irradiated with 1 Gy at 6 MV; M ± SEM–mean ± standard error of the mean. (DOCX) [file pone.0253553.s004.docx]

**S4 Table. Statistical characteristics of the cell count of the A549 lung carcinoma epithelial cells treated with Cu(Picolinyl-L-Tyrosinate)_2._** Ctrl/PBS – non-irradiated cells with PBS; kV/PBS – cells with PBS irradiated with 1 Gy at 120 kV; MV/PBS - cells with PBS irradiated with 1 Gy at 6 MV; Ctrl/CuPLTyr-10μM - non-irradiated cells treated with 10 μM Cu(Picolinyl-L-Tyrosinate)_2_; kV/CuPLTyr-10μM - cells treated with 10 μM Cu(Picolinyl-L-Tyrosinate)_2_ and irradiated with 1 Gy at 120 kV; MV/CuPLTyr-10μM - cells treated with 10 μM Cu(Picolinyl-L-Tyrosinate)_2_ and irradiated with 1 Gy at 6 MV; Ctrl/CuPLTyr-100μM - non-irradiated cells treated with 100 μM Cu(Picolinyl-L-Tyrosinate)_2_; kV/CuPLTyr-100μM - cells treated with 100 μM Cu(Picolinyl-L-Tyrosinate)_2_ and irradiated with 1 Gy at 120 kV; MV/CuPLTyr-100μM - cells treated with 100 μM Cu(Picolinyl-L-Tyrosinate)_2_ and irradiated with 1 Gy at 6 MV; *M ± SEM – mean ± standard error of the mean.*

| **Group** | **Days** | **Мean ± SEM** | **Compared groups** | **Difference (times)** | ***P*** |
| --- | --- | --- | --- | --- | --- |
| **Ctrl/CuPLTyr-10μM** | **Day 8** | 311125 ± 11025 | Ctrl/CuPLTyr-10μM vs. Ctrl/PBS | 3.5 | < 0.0001 |
|  |  |  | Ctrl/CuPLTyr-10μM vs. Ctrl/CuPLTyr-100μM | 17 | < 0.0001 |
|  |  |  | Ctrl/CuPLTyr-10μM vs. kV/CuPLTyr-10μM | 3.4 | < 0.0001 |
| **kV/CuPLTyr-10μM** | **Day 4** | 19350 ± 600 | kV/CuPLTyr-10μM vs. kV/PBS | 7.7 | < 0.05 |
|  | **Day 8** | 89775 ± 15075 | kV/CuPLTyr-10μM vs. kV/PBS | 9.4 | < 0.0001 |
|  |  |  | kV/CuPLTyr-10μM vs. MV/CuPLTyr-10μM | 4.3 | < 0.0001 |
|  |  |  | kV/CuPLTyr-10μM vs. kV/CuPLTyr-100μM | 11 | < 0.05 |
| **MV/CuPLTyr-10μM** | **Day 8** | 387725 ± 37675 | MV/CuPLTyr-10μM vs. MV/PBS | 2 | < 0.0001 |
|  |  |  | MV/CuPLTyr-10μM vs. MV/CuPLTyr-100μM | 43.4 | < 0.0001 |
| **Ctrl/CuPLTyr-100μM** | **Day 4** | 12775 ± 275 | Ctrl/CuPLTyr-100μM vs. Ctrl/PBS | 1.03 | < 0.01 |
|  | **Day 8** | 18175 ± 625 | Ctrl/CuPLTyr-100μM vs. Ctrl/PBS | 60 | < 0.0001 |
| **kV/CuPLTyr-100μM** | **Day 4** | 6475 ± 1025 | kV/CuPLTyr-100μM vs. kV/PBS | 23 | < 0.01 |
|  | **Day 8** | 8050 ± 850 | kV/CuPLTyr-100μM vs. kV/PBS | 105 | < 0.0001 |
| **MV/CuPLTyr-100μM** | **Day 8** | 8925 ± 5125 | MV/CuPLTyr-100μM vs. MV/PBS | 84 | < 0.0001 |
