# Supplementary material for: The radioenhancement potential of Schiff base derived copper (II) compounds against lung carcinoma in vitro
Source: PLoS One. 2021 Jun 18;16(6):e0253553. doi: 10.1371/journal.pone.0253553 (PMC8213134; doi:10.1371/journal.pone.0253553)
Supplement: S5 Table — Ctrl/PBS–non-irradiated cells with PBS; kV/PBS–cells with PBS irradiated with 1 Gy at 120 kV; MV/PBS—cells with PBS irradiated with 1 Gy at 6 MV; Ctrl/CuPLTyr-10μM—non-irradiated cells treated with 10 μM Cu(Picolinyl-L-Tyrosinate)2; kV/CuPLTyr-10μM—cells treated with 10 μM Cu(Picolinyl-L-Tyrosinate)2 and irradiated with 1 Gy at 120 kV; MV/CuPLTyr-10μM—cells treated with 10 μM Cu(Picolinyl-L-Tyrosinate)2 and irradiated with 1 Gy at 6 MV; Ctrl/CuPLTyr-100μM—non-irradiated cells treated with 100 μM Cu(Picolinyl-L-Tyrosinate)2; kV/CuPLTyr-100μM—cells treated with 100 μM Cu(Picolinyl-L-Tyrosinate)2 and irradiated with 1 Gy at 120 kV; MV/CuPLTyr-100μM—cells treated with 100 μM Cu(Picolinyl-L-Tyrosinate)2 and irradiated with 1 Gy at 6 MV; M ± SEM–mean ± standard error of the mean. (DOCX) [file pone.0253553.s005.docx]

**S5 Table. Statistical characteristics of the WST-1 cell viability assay of the cells treated CuPLTyr.** Ctrl/PBS – non-irradiated cells with PBS; kV/PBS – cells with PBS irradiated with 1 Gy at 120 kV; MV/PBS - cells with PBS irradiated with 1 Gy at 6 MV; Ctrl/CuPLTyr-10μM - non-irradiated cells treated with 10 μM Cu(Picolinyl-L-Tyrosinate)_2_; kV/CuPLTyr-10μM - cells treated with 10 μM Cu(Picolinyl-L-Tyrosinate)_2_ and irradiated with 1 Gy at 120 kV; MV/CuPLTyr-10μM - cells treated with 10 μM Cu(Picolinyl-L-Tyrosinate)_2_ and irradiated with 1 Gy at 6 MV; Ctrl/CuPLTyr-100μM - non-irradiated cells treated with 100 μM Cu(Picolinyl-L-Tyrosinate)_2_; kV/CuPLTyr-100μM - cells treated with 100 μM Cu(Picolinyl-L-Tyrosinate)_2_ and irradiated with 1 Gy at 120 kV; MV/CuPLTyr-100μM - cells treated with 100 μM Cu(Picolinyl-L-Tyrosinate)_2_ and irradiated with 1 Gy at 6 MV; *M ± SEM – mean ± standard error of the mean.*

| **Group** | **М±SEM** | **Compared groups** | **Difference (times)** | ***P*** |
| --- | --- | --- | --- | --- |
| **Ctrl/CuPLTyr-10μM** | 0.062 ± 0.005 | Ctrl/CuPLTyr-10μM vs. Ctrl/PBS | 2 | < 0.05 |
| **kV/CuPLTyr-10μM** | 0.037 ± 0.008 | kV/CuPLTyr-10μM vs. kV/PBS | 3 | < 0.05 |
|  |  | kV/CuPLTyr-10μM vs. MV/CuPLTyr-10μM | 3 | < 0.05 |
| **MV/CuPLTyr-/10μM** | 0.110 ± 0.031 | MV/CuPLTyr-10μM vs. MV/CuPLTyr-100μM | 15.7 | < 0.01 |
| **Ctrl/CuPLTyr -100μM** | 0.055 ± 0.024 | Ctrl/CuPLTyr-100μM vs. Ctrl/PBS | 2.3 | < 0.05 |
| **kV/CuPLTyr-100μM** | 0.013 ± 0.001 | kV/CuPLTyr-100μM vs. kV/PBS | 8 | < 0.05 |
| **MV/CuPLTyr-100μM** | 0.007 ± 0.002 | MV/CuPLTyr-100μM vs. MV/PBS | 17 | < 0.01 |
