# Supplementary material for: The radioenhancement potential of Schiff base derived copper (II) compounds against lung carcinoma in vitro
Source: PLoS One. 2021 Jun 18;16(6):e0253553. doi: 10.1371/journal.pone.0253553 (PMC8213134; doi:10.1371/journal.pone.0253553)
Supplement: S6 Table — Ctrl/CuPLTyr-10μM—non-irradiated cells treated with 10 μM Cu(Picolinyl-L-Tyrosinate)2; Ctrl/CuPLTyr-100μM—non-irradiated cells treated with 100 μM Cu(Picolinyl-L-Tyrosinate)2; M ± SEM–mean ± standard error of the mean. (DOCX) [file pone.0253553.s006.docx]

**S6 Table. Statistical characteristics of the BrdU cell proliferation assay of the cells treated with CuPLTyr.** Ctrl/CuPLTyr-10μM - non-irradiated cells treated with 10 μM Cu(Picolinyl-L-Tyrosinate)_2_; Ctrl/CuPLTyr-100μM - non-irradiated cells treated with 100 μM Cu(Picolinyl-L-Tyrosinate)_2_; *M ± SEM – mean ± standard error of the mean.*

| **Group** | **М±SEM** | **Compared groups** | **Difference (times)** | ***P*** |
| --- | --- | --- | --- | --- |
| **Ctrl/CuPLTyr-10μM** | 0.261 ± 0.013 | Ctrl/CuPLTyr-10μM vs. Ctrl/CuPLTyr-100μM | 1.2 | < 0.01 |
| **Ctrl/CuPLTyr-100μM** | 0.222 ± 0.004 |  |  |  |
