# Supplementary material for: The radioenhancement potential of Schiff base derived copper (II) compounds against lung carcinoma in vitro
Source: PLoS One. 2021 Jun 18;16(6):e0253553. doi: 10.1371/journal.pone.0253553 (PMC8213134; doi:10.1371/journal.pone.0253553)
Supplement: S8 Table — kV/PBS–cells with PBS irradiated with 1 Gy at 120 kV; MV/PBS—cells with PBS irradiated with 1 Gy at 6 MV; kV/CuILTyr-10μM—cells treated with 10 μM Cu(Isonicotinyl-L-Tyrosinate)2 and irradiated with 1 Gy at 120 kV; MV/CuILTyr-10μM—cells treated with 10 μM Cu(Isonicotinyl-L-Tyrosinate)2 and irradiated with 1 Gy at 6 MV; Ctrl/CuILTyr-100μM—non-irradiated cells treated with 100 μM Cu(Isonicotinyl-L-Tyrosinate)2; kV/CuILTyr-100μM—cells treated with 100 μM Cu(Isonicotinyl-L-Tyrosinate)2 and irradiated with 1 Gy at 120 kV; MV/CuILTyr-100μM—cells treated with 100 μM Cu(Isonicotinyl-L-Tyrosinate)2 and irradiated with 1Gy at 6 MV; M ± SEM–mean ± standard error of the mean. (DOCX) [file pone.0253553.s008.docx]

**S8 Table. Statistical characteristics of the WST-1 cell viability assay of the cells treated with CuILTyr.** kV/PBS – cells with PBS irradiated with 1 Gy at 120 kV; MV/PBS - cells with PBS irradiated with 1 Gy at 6 MV; kV/CuILTyr-10μM - cells treated with 10 μM Cu(Isonicotinyl-L-Tyrosinate)_2_ and irradiated with 1 Gy at 120 kV; MV/CuILTyr-10μM - cells treated with 10 μM Cu(Isonicotinyl-L-Tyrosinate)_2_ and irradiated with 1 Gy at 6 MV; Ctrl/CuILTyr-100μM - non-irradiated cells treated with 100 μM Cu(Isonicotinyl-L-Tyrosinate)_2_; kV/CuILTyr-100μM - cells treated with 100 μM Cu(Isonicotinyl-L-Tyrosinate)_2_ and irradiated with 1 Gy at 120 kV; MV/CuILTyr-100μM - cells treated with 100 μM Cu(Isonicotinyl-L-Tyrosinate)_2_ and irradiated with 1Gy at 6 MV; *M ± SEM – mean ± standard error of the mean*.

| **Group** | **М±SEM** | **Compared groups** | **Difference (times)** | ***P*** |
| --- | --- | --- | --- | --- |
| **kV/CuILTyr-10μM** | 0.100 ± 0.018 | kV/CuILTyr-10μM vs. kV/CuILTyr-100μM | 2 | < 0.01 |
| **MV/CuILTyr-10μM** | 0.099 ± 0.013 | MV/CuILTyr-10μM vs. MV/CuILTyr-100μM | 2 | < 0.05 |
| **Ctrl/CuILTyr-100μM** | 0.101 ± 0.010 | Ctrl/CuILTyr-100μM vs. kV/CuILTyr-100μM | 2 | < 0.01 |
|  |  | Ctrl/CuILTyr-100μM vs. MV/CuILTyr-100μM | 2 | < 0.05 |
| **kV/CuILTyr-100μM** | 0.050 ± 0.012 | kV/CuILTyr-100μM vs. kV/PBS | 2 | < 0.01 |
| **MV/CuILTyr-100μM** | 0.052 ± 0.009 | MV/CuILTyr-100μM vs. MV/PBS | 2.3 | < 0.05 |
