# Supplementary material for: The radioenhancement potential of Schiff base derived copper (II) compounds against lung carcinoma in vitro
Source: PLoS One. 2021 Jun 18;16(6):e0253553. doi: 10.1371/journal.pone.0253553 (PMC8213134; doi:10.1371/journal.pone.0253553)
Supplement: S9 Table — Ctrl/PBS–non-irradiated cells with PBS; kV/PBS–cells with PBS irradiated with 1 Gy at 120 kV; MV/PBS—cells with PBS irradiated with 1 Gy at 6 MV; Ctrl/CuPLTrp-10μM—non-irradiated cells treated with 10 μM Cu(Picolinyl-L-Tryptophanate)2; kV/CuPLTrp-10μM—cells treated with 10 μM Cu(Picolinyl-L-Tryptophanate)2 and irradiated with 1 Gy at 120 kV; MV/CuPLTrp-10μM—cells treated with 10 μM Cu(Picolinyl-L-Tryptophanate)2 and irradiated with 1 Gy at 6 MV; Ctrl/CuPLTrp-100μM—non-irradiated cells treated with 100 μM Cu(Picolinyl-L-Tryptophanate)2; kV/CuPLTrp-100μM—cells treated with 100 μM Cu(Picolinyl-L-Tryptophanate)2 and irradiated with 1 Gy at 120 kV; MV/CuPLTrp-100μM—cells treated with 100 μM Cu(Picolinyl-L-Tryptophanate)2 and irradiated with 1 Gy at 6 MV; M ± SEM–mean ± standard error of the mean. (DOCX) [file pone.0253553.s009.docx]

**S9 Table. Statistical characteristics of the cell count of the A549 lung carcinoma epithelial cells treated with Cu(Picolinyl-L-Tryptophanate)_2._** Ctrl/PBS – non-irradiated cells with PBS; kV/PBS – cells with PBS irradiated with 1 Gy at 120 kV; MV/PBS - cells with PBS irradiated with 1 Gy at 6 MV; Ctrl/CuPLTrp-10μM - non-irradiated cells treated with 10 μM Cu(Picolinyl-L-Tryptophanate)_2_; kV/CuPLTrp-10μM - cells treated with 10 μM Cu(Picolinyl-L-Tryptophanate)_2_ and irradiated with 1 Gy at 120 kV; MV/CuPLTrp-10μM - cells treated with 10 μM Cu(Picolinyl-L-Tryptophanate)_2_ and irradiated with 1 Gy at 6 MV; Ctrl/CuPLTrp-100μM - non-irradiated cells treated with 100 μM Cu(Picolinyl-L-Tryptophanate)_2_; kV/CuPLTrp-100μM - cells treated with 100 μM Cu(Picolinyl-L-Tryptophanate)_2_ and irradiated with 1 Gy at 120 kV; MV/CuPLTrp-100μM - cells treated with 100 μM Cu(Picolinyl-L-Tryptophanate)_2_ and irradiated with 1 Gy at 6 MV; *M ± SEM – mean ± standard error of the mean*.

| **Group** | **Days** | **Мean ± SEM** | **Compared groups** | **Difference (times)** | ***P*** |
| --- | --- | --- | --- | --- | --- |
| **Ctrl/CuPLTrp-10μM** | **Day 4** | 115525 ± 5225 | Ctrl/CuPLTrp-10μM vs. Ctrl/CuPLTrp-100μM | 6.2 | < 0.05 |
|  | **Day 8** | 1118250 ± 57750 | Ctrl/CuPLTrp-10μM vs. kV/CuPLTrp-10μM | 2.1 | < 0.0001 |
|  |  |  | Ctrl/CuPLTrp-10μM vs. MV/CuPLTrp-10μM | 1.5 | < 0.0001 |
|  |  |  | Ctrl/CuPLTrp-10μM vs. Ctrl/CuPLTrp-100μM | 44 | < 0.0001 |
| **kV/CuPLTrp-10μM** | **Day 4** | 55300 ± 3850 | kV/CuPLTrp-10μM vs. kV/PBS | 2.7 | <0.05 |
|  | **Day 8** | 535750 ± 750 | kV/CuPLTrp-10μM vs. kV/PBS | 1.6 | <0.0001 |
|  |  |  | kV/CuPLTrp-10μM vs. MV/CuPLTrp-10μM | 1.4 | <0.0001 |
|  |  |  | kV/CuPLTrp-10μM vs. kV/CuPLTrp-100μM | 33 | <0.0001 |
| **MV/CuPLTrp-10μM** | **Day 8** | 758750 ± 14250 | MV/CuPLTrp-10μM vs. MV/CuPLTrp-100μM | 27 | < 0.0001 |
| **Ctrl/CuPLTrp-100μM** | **Day 4** | 18525 ± 125 | Ctrl/CuPLTrp-100μM vs. Ctrl/PBS | 7 | < 0.01 |
|  | **Day 8** | 25425 ± 8125 | Ctrl/CuPLTrp-100μM vs. Ctrl/PBS | 43 | < 0.0001 |
| **kV/CuPLTrp-100μM** | **Day 4** | 6175 ± 775 | kV/CuPLTrp-100μM vs. kV/PBS | 24 | <0.0001 |
|  | **Day 8** | 16475 ± 5725 | kV/CuPLTrp-100μM vs. kV/PBS | 51 | <0.0001 |
| **MV/CuPLTrp-100μM** | **Day 8** | 28075 ± 1775 | MV/CuPLTrp-100μM vs. MV/PBS | 27 | < 0.0001 |
