# Supplementary material for: The radioenhancement potential of Schiff base derived copper (II) compounds against lung carcinoma in vitro
Source: PLoS One. 2021 Jun 18;16(6):e0253553. doi: 10.1371/journal.pone.0253553 (PMC8213134; doi:10.1371/journal.pone.0253553)
Supplement: S11 Table — MV/CuPLTrp-10μM—cells treated with 10 μM Cu(Picolinyl-L-Tryptophanate)2 and irradiated with 1 Gy at 6 MV; MV/CuPLTrp-100μM—cells treated with 100 μM Cu(Picolinyl-L-Tryptophanate)2 and irradiated with 1 Gy at 6 MV; M ± SEM–mean ± standard error of the mean. (DOCX) [file pone.0253553.s011.docx]

**S11 Table. Statistical characteristics of the BrdU cell proliferation assay of the cells treated with CuPLTrp with PBS and irradiated with 1 Gy at 6 MV vs. non-irradiated controls.** MV/CuPLTrp-10μM - cells treated with 10 μM Cu(Picolinyl-L-Tryptophanate)_2_ and irradiated with 1 Gy at 6 MV; MV/CuPLTrp-100μM - cells treated with 100 μM Cu(Picolinyl-L-Tryptophanate)_2_ and irradiated with 1 Gy at 6 MV; *M ± SEM – mean ± standard error of the mean*.

| **Group** | **М±SEM** | **Compared groups** | **Difference (times)** | ***P*** |
| --- | --- | --- | --- | --- |
| **MV/CuPLTrp-10μM** | 0.306 ± 0.027 | MV/CuPLTrp-10μM vs. MV/CuPLTrp-100μM | 1.4 | < 0.05 |
| **MV/CuPLTrp-100μM** | 0.226 ± 0.004 |  |  |  |
