# Supplementary material for: The radioenhancement potential of Schiff base derived copper (II) compounds against lung carcinoma in vitro
Source: PLoS One. 2021 Jun 18;16(6):e0253553. doi: 10.1371/journal.pone.0253553 (PMC8213134; doi:10.1371/journal.pone.0253553)
Supplement: S12 Table — Ctrl/PBS–non-irradiated cells with PBS; kV/PBS–cells with PBS irradiated with 1 Gy at 120 kV; MV/PBS—cells with PBS irradiated with 1 Gy at 6 MV; Ctrl/CuPLPhe-10μM—non-irradiated cells treated with 10 μM Cu(Picolinyl-L- Phenylalaninate)2; kV/CuPLPhe-10μM—cells treated with 10 μM Cu(Picolinyl-L- Phenylalaninate)2 and irradiated with 1 Gy at 120 kV; MV/CuPLPhe-10μM—cells treated with 10 μM Cu(Picolinyl-L- Phenylalaninate)2 and irradiated with 1 Gy at 6 MV; Ctrl/CuPLPhe-100μM—non-irradiated cells treated with 100 μM Cu(Picolinyl-L- Phenylalaninate)2; kV/CuPLPhe-100μM—cells treated with 100 μM Cu(Picolinyl-L- Phenylalaninate)2 and irradiated with 1 Gy at 120 kV; MV/CuPLPhe-100μM—cells treated with 100 μM Cu(Picolinyl-L-Phenylalaninate)2 and irradiated with 1 Gy at 6 MV; M ± SEM–mean ± standard error of the mean. (DOCX) [file pone.0253553.s012.docx]

**S12 Table. Statistical characteristics of the cell count of the A549 lung carcinoma epithelial cells treated with Cu(Picolinyl-L-Phenylalaninate)_2._** Ctrl/PBS – non-irradiated cells with PBS; kV/PBS – cells with PBS irradiated with 1 Gy at 120 kV; MV/PBS - cells with PBS irradiated with 1 Gy at 6 MV; Ctrl/CuPLPhe-10μM - non-irradiated cells treated with 10 μM Cu(Picolinyl-L- Phenylalaninate)_2_; kV/CuPLPhe-10μM - cells treated with 10 μM Cu(Picolinyl-L- Phenylalaninate)_2_ and irradiated with 1 Gy at 120 kV; MV/CuPLPhe-10μM - cells treated with 10 μM Cu(Picolinyl-L- Phenylalaninate)_2_ and irradiated with 1 Gy at 6 MV; Ctrl/CuPLPhe-100μM - non-irradiated cells treated with 100 μM Cu(Picolinyl-L- Phenylalaninate)_2_; kV/CuPLPhe-100μM - cells treated with 100 μM Cu(Picolinyl-L- Phenylalaninate)_2_ and irradiated with 1 Gy at 120 kV; MV/CuPLPhe-100μM - cells treated with 100 μM Cu(Picolinyl-L-Phenylalaninate)_2_ and irradiated with 1 Gy at 6 MV; *M ± SEM – mean ± standard error of the mean*.

| **Group** | **Days** | **Мean ± SEM** | **Compared groups** | **Difference (times)** | ***P*** |
| --- | --- | --- | --- | --- | --- |
| **Ctrl/CuPLPhe-10μM** | **Day 8** | 97475 ± 2825 | Ctrl/CuPLPhe-10μM vs. Ctrl/PBS | 11 | < 0.0001 |
|  |  |  | Ctrl/CuPLPhe-10μM vs. kV/CuPLPhe-10μM | 4 | < 0.0001 |
| **kV/CuPLPhe-10μM** | **Day 8** | 383700 ± 114900 | kV/CuPLPhe-10μM vs. kV/PBS | 2.2 | < 0.0001 |
|  |  |  | kV/CuPLPhe-10μM vs. MV/CuPLPhe-10μM | 6.5 | < 0.0001 |
|  |  |  | kV/CuPLPhe-10μM vs. kV/CuPLPhe-100μM | 1.6 | < 0.05 |
| **MV/CuPLPhe-10μM** | **Day 8** | 59425 ± 15175 | MV/CuPLPhe-10μM vs. MV/PBS | 13 | < 0.0001 |
| **Ctrl/CuPLPhe-100μM** | **Day 8** | 10325 ± 325 | Ctrl/CuPLPhe-100μM vs. Ctrl/PBS | 106 | < 0.0001 |
|  |  |  | Ctrl/CuPLPhe-100μM vs. kV/CuPLPhe-100μM | 23 | < 0.0001 |
| **kV/CuPLPhe-100μM** | **Day 8** | 237650 ± 17450 | kV/CuPLPhe-100μM vs. kV/PBS | 4 | < 0.0001 |
|  |  |  | kV/CuPLPhe-100μM vs. MV/CuPLPhe-100μM | 33 | < 0.0001 |
| **MV/CuPLPhe-100μM** | **Day 8** | 7150 ± 1800 | MV/CuPLPhe-100μM vs. MV/PBS | 105 | < 0.0001 |
