# Supplementary material for: The radioenhancement potential of Schiff base derived copper (II) compounds against lung carcinoma in vitro
Source: PLoS One. 2021 Jun 18;16(6):e0253553. doi: 10.1371/journal.pone.0253553 (PMC8213134; doi:10.1371/journal.pone.0253553)
Supplement: S13 Table — Ctrl/PBS–non-irradiated cells with PBS; kV/PBS–cells with PBS irradiated with 1 Gy at 120 kV; MV/PBS—cells with PBS irradiated with 1 Gy at 6 MV; Ctrl/CuPLPhe-10μM—non-irradiated cells treated with 10 μM Cu(Picolinyl-L- Phenylalaninate)2; kV/CuPLPhe-10μM—cells treated with 10 μM Cu(Picolinyl-L- Phenylalaninate)2 and irradiated with 1 Gy at 120 kV; MV/CuPLPhe-10μM—cells treated with 10 μM Cu(Picolinyl-L- Phenylalaninate)2 and irradiated with 1 Gy at 6 MV; Ctrl/CuPLPhe-100μM—non-irradiated cells treated with 100 μM Cu(Picolinyl-L- Phenylalaninate)2; kV/CuPLPhe-100μM—cells treated with 100 μM Cu(Picolinyl-L- Phenylalaninate)2 and irradiated with 1 Gy at 120 kV; MV/CuPLPhe-100μM—cells treated with 100 μM Cu(Picolinyl-L-Phenylalaninate)2 and irradiated with 1 Gy at 6 MV; M ± SEM–mean ± standard error of the mean. (DOCX) [file pone.0253553.s013.docx]

**S13 Table. Statistical characteristics of the WST-1 cell viability assay of the cells exposed to CuPLPhe with PBS and irradiated with 1 Gy at either 120 kV or 6 MV vs. non-irradiated controls.** Ctrl/PBS – non-irradiated cells with PBS; kV/PBS – cells with PBS irradiated with 1 Gy at 120 kV; MV/PBS - cells with PBS irradiated with 1 Gy at 6 MV; Ctrl/CuPLPhe-10μM - non-irradiated cells treated with 10 μM Cu(Picolinyl-L- Phenylalaninate)_2_; kV/CuPLPhe-10μM - cells treated with 10 μM Cu(Picolinyl-L- Phenylalaninate)_2_ and irradiated with 1 Gy at 120 kV; MV/CuPLPhe-10μM - cells treated with 10 μM Cu(Picolinyl-L- Phenylalaninate)_2_ and irradiated with 1 Gy at 6 MV; Ctrl/CuPLPhe-100μM - non-irradiated cells treated with 100 μM Cu(Picolinyl-L- Phenylalaninate)_2_; kV/CuPLPhe-100μM - cells treated with 100 μM Cu(Picolinyl-L- Phenylalaninate)_2_ and irradiated with 1 Gy at 120 kV; MV/CuPLPhe-100μM - cells treated with 100 μM Cu(Picolinyl-L-Phenylalaninate)_2_ and irradiated with 1 Gy at 6 MV; *M ± SEM – mean ± standard error of the mean*.

| **Group** | **М±SEM** | **Compared groups** | **Difference (times)** | ***P*** |
| --- | --- | --- | --- | --- |
| **Ctrl/CuPLPhe-10μM** | 0.127 ± 0.019 | Ctrl/CuPLPhe-10μM vs. Ctrl/CuPLPhe-100μM | 7.5 | < 0.0001 |
|  |  | Ctrl/CuPLPhe-10μM vs. kV/CuPLPhe-10μM | 3 | < 0.0001 |
|  |  | Ctrl/CuPLPhe-10μM vs. MV/CuPLPhe-10μM | 2 | < 0.0001 |
| **kV/CuPLPhe-10μM** | 0.044 ± 0.006 | kV/CuPLPhe-10μM vs. kV/PBS | 2.3 | < 0.01 |
| **MV/CuPLPhe-10μM** | 0.064 ± 0.010 | MV/CuPLPhe-10μM vs. MV/PBS | 2 | < 0.01 |
|  |  | MV/CuPLPhe-10μM vs. MV/CuPLPhe-100μM | 5 | < 0.01 |
| **Ctrl/CuPLPhe-100μM** | 0.017 ± 0.003 | Ctrl/CuPLPhe-100μM vs. Ctrl/PBS | 7.4 | < 0.0001 |
| **kV/CuPLPhe-100μM** | 0.009 ± 0.002 | kV/CuPLPhe-100μM vs. kV/PBS | 11.4 | < 0.0001 |
| **MV/CuPLPhe-100μM** | 0.013 ± 0.004 | MV/CuPLPhe-100μM vs. MV/PBS | 9 | < 0.0001 |
