# Supplementary material for: The radioenhancement potential of Schiff base derived copper (II) compounds against lung carcinoma in vitro
Source: PLoS One. 2021 Jun 18;16(6):e0253553. doi: 10.1371/journal.pone.0253553 (PMC8213134; doi:10.1371/journal.pone.0253553)
Supplement: S14 Table — Ctrl/PBS–non-irradiated cells with PBS; kV/PBS–cells with PBS irradiated with 1 Gy at 120 kV; MV/PBS—cells with PBS irradiated with 1 Gy at 6 MV; Ctrl/CuNLPhe-10μM—non-irradiated cells treated with 10 μM Cu(Nicotinyl-L- Phenylalaninate)2; kV/CuNLPhe-10μM—cells treated with 10 μM Cu(Nicotinyl-L- Phenylalaninate)2 and irradiated with 1Gy at 120 kV; MV/CuNLPhe-10μM—cells treated with 10 μM Cu(Nicotinyl-L- Phenylalaninate)2 and irradiated with 1Gy at 6 MV; Ctrl/CuNLPhe-100μM—non-irradiated cells treated with 100 μM Cu(Nicotinyl-L- Phenylalaninate)2; kV/CuNLPhe-100μM—cells treated with 100 μM Cu(Nicotinyl-L- Phenylalaninate)2 and irradiated with 1 Gy at 120 kV; MV/CuNLPhe-100μM—cells treated with 100 μM Cu(Nicotinyl-L-Phenylalaninate)2 and irradiated with 1 Gy at 6 MV; M ± SEM–mean ± standard error of the mean. (DOCX) [file pone.0253553.s014.docx]

**S14 Table. Statistical characteristics of the cell count of the A549 lung carcinoma epithelial cells treated with Cu(Nicotinyl-L-Phenylalaninate)_2._** Ctrl/PBS – non-irradiated cells with PBS; kV/PBS – cells with PBS irradiated with 1 Gy at 120 kV; MV/PBS - cells with PBS irradiated with 1 Gy at 6 MV; Ctrl/CuNLPhe-10μM - non-irradiated cells treated with 10 μM Cu(Nicotinyl-L- Phenylalaninate)_2_; kV/CuNLPhe-10μM - cells treated with 10 μM Cu(Nicotinyl-L- Phenylalaninate)_2_ and irradiated with 1Gy at 120 kV; MV/CuNLPhe-10μM - cells treated with 10 μM Cu(Nicotinyl-L- Phenylalaninate)_2_ and irradiated with 1Gy at 6 MV; Ctrl/CuNLPhe-100μM - non-irradiated cells treated with 100 μM Cu(Nicotinyl-L- Phenylalaninate)_2_; kV/CuNLPhe-100μM - cells treated with 100 μM Cu(Nicotinyl-L- Phenylalaninate)_2_ and irradiated with 1 Gy at 120 kV; MV/CuNLPhe-100μM - cells treated with 100 μM Cu(Nicotinyl-L-Phenylalaninate)_2_ and irradiated with 1 Gy at 6 MV; *M ± SEM – mean ± standard error of the mean*.

| **Group** | **Days** | **Мean ± SEM** | **Compared groups** | **Difference (times)** | ***P*** |
| --- | --- | --- | --- | --- | --- |
| **Ctrl/CuNLPhe-10μM** | **Day 8** | 1005750 ± 76250 | Ctrl/CuNLPhe-10μM vs. Ctrl/PBS | 1.1 | < 0.05 |
|  |  |  | Ctrl/CuNLPhe-10μM vs. kV/CuNLPhe-10μM | 2.4 | < 0.0001 |
|  |  |  | Ctrl/CuNLPhe-10μM vs. MV/CuNLPhe-10μM | 1.5 | < 0.0001 |
|  |  |  | Ctrl/CuNLPhe-10μM vs. Ctrl/CuNLPhe-100μM | 2.3 | < 0.0001 |
| **kV/CuNLPhe-10μM** | **Day 4** | 16925 ± 75 | kV/CuNLPhe-10μM vs. kV/PBS | 8.8 | < 0.05 |
|  | **Day 8** | 413675 ± 44175 | kV/CuNLPhe-10μM vs. kV/PBS | 2 | < 0.0001 |
|  |  |  | kV/CuNLPhe-10μM vs. MV/CuNLPhe-10μM | 1.6 | < 0.0001 |
|  |  |  | kV/CuNLPhe-10μM vs. kV/CuNLPhe-100μM | 1.7 | < 0.001 |
| **MV/CuNLPhe-10μM** | **Day 8** | 666250 ± 42250 | MV/CuNLPhe-10μM vs. MV/PBS |  | < 0.05 |
|  |  |  | MV/CuNLPhe-10μM vs. MV/CuNLPhe-100μM | 2 | < 0.0001 |
| **Ctrl/CuNLPhe-100μM** | **Day 8** | 429300 ± 36450 | Ctrl/CuNLPhe-100μM vs. Ctrl/PBS | 2.6 | < 0.0001 |
|  |  |  | Ctrl/CuNLPhe-100μM vs. kV/CuNLPhe-100μM | 1.8 | < 0.0001 |
|  |  |  | Ctrl/CuNLPhe-100μM vs. MV/CuNLPhe-100μM |  | < 0.05 |
| **kV/CuNLPhe-100μM** | **Day 4** | 5875 ± 25 | kV/CuNLPhe-100μM vs. kV/PBS | 25 | < 0.01 |
|  | **Day 8** | 244100 ± 1800 | kV/CuNLPhe-100μM vs. kV/PBS | 3.5 | < 0.0001 |
|  |  |  | kV/CuNLPhe-100μM vs. MV/CuNLPhe-100μM |  | < 0.05 |
| **MV/CuNLPhe-100μM** | **Day 8** | 331275 ± 11375 | MV/CuNLPhe-100μM vs. MV/PBS | 2.3 | < 0.0001 |
