# Supplementary material for: The radioenhancement potential of Schiff base derived copper (II) compounds against lung carcinoma in vitro
Source: PLoS One. 2021 Jun 18;16(6):e0253553. doi: 10.1371/journal.pone.0253553 (PMC8213134; doi:10.1371/journal.pone.0253553)
Supplement: S15 Table — kV/PBS–cells only with PBS irradiated with 1 Gy at 120 kV; MV/PBS—cells with PBS irradiated with 1 Gy at 6 MV; Ctrl/CuNLPhe-10μM—non-irradiated cells treated with 10 μM Cu(Nicotinyl-L- Phenylalaninate)2; kV/CuNLPhe-10μM—cells treated with 10 μM Cu(Nicotinyl-L- Phenylalaninate)2 and irradiated with 1Gy at 120 kV; MV/CuNLPhe-10μM—cells treated with 10 μM Cu(Nicotinyl-L- Phenylalaninate)2 and irradiated with 1Gy at 6 MV; Ctrl/CuNLPhe-100μM—non-irradiated cells treated with 100 μM Cu(Nicotinyl-L- Phenylalaninate)2; kV/CuNLPhe-100μM—cells treated with 100 μM Cu(Nicotinyl-L- Phenylalaninate)2 and irradiated with 1 Gy at 120 kV; MV/CuNLPhe-100μM—cells treated with 100 μM Cu(Nicotinyl-L-Phenylalaninate)2 and irradiated with 1 Gy at 6 MV; M ± SEM–mean ± standard error of the mean. (DOCX) [file pone.0253553.s015.docx]

**S15 Table. Statistical characteristics of the WST-1 cell viability assay of the cells exposed to CuNLPhe with PBS and irradiated with 1 Gy at either 120 kV or 6 MV vs. non-irradiated controls.** kV/PBS – cells only with PBS irradiated with 1 Gy at 120 kV; MV/PBS - cells with PBS irradiated with 1 Gy at 6 MV; Ctrl/CuNLPhe-10μM - non-irradiated cells treated with 10 μM Cu(Nicotinyl-L- Phenylalaninate)_2_; kV/CuNLPhe-10μM - cells treated with 10 μM Cu(Nicotinyl-L- Phenylalaninate)_2_ and irradiated with 1Gy at 120 kV; MV/CuNLPhe-10μM - cells treated with 10 μM Cu(Nicotinyl-L- Phenylalaninate)_2_ and irradiated with 1Gy at 6 MV; Ctrl/CuNLPhe-100μM - non-irradiated cells treated with 100 μM Cu(Nicotinyl-L- Phenylalaninate)_2_; kV/CuNLPhe-100μM - cells treated with 100 μM Cu(Nicotinyl-L- Phenylalaninate)_2_ and irradiated with 1 Gy at 120 kV; MV/CuNLPhe-100μM - cells treated with 100 μM Cu(Nicotinyl-L-Phenylalaninate)_2_ and irradiated with 1 Gy at 6 MV; *M ± SEM – mean ± standard error of the mean*.

| **Group** | **М±SEM** | **Compared groups** | **Difference (times)** | ***P*** |
| --- | --- | --- | --- | --- |
| **Ctrl/CuNLPhe-10μM** | 0.180 ± 0.027 | Ctrl/CuNLPhe-10μM vs. Ctrl/CuNLPhe-100μM | 1.7 | < 0.01 |
|  |  | Ctrl/CuNLPhe-10μM vs. MV/CuNLPhe-10μM | 1.8 | <0.01 |
| **kV/CuNLPhe-10μM** | 0.135 ± 0.024 | kV/CuNLPhe-10μM vs. kV/CuNLPhe-100μM | 3.5 | < 0.0001 |
| **MV/CuNLPhe-10μM** | 0.102 ± 0.010 | MV/CuNLPhe-10μM vs. MV/CuNLPhe-100μM | 3 | < 0.01 |
| **Ctrl/CuNLPhe-100μM** | 0.104 ± 0.014 | Ctrl/CuNLPhe-100μM vs. kV/CuNLPhe-100μM | 2.7 | < 0.05 |
|  |  | Ctrl/CuNLPhe-100μM vs. MV/CuNLPhe-100μM | 3 | < 0.01 |
| **kV/CuNLPhe-100μM** | 0.039 ± 0.011 | kV/CuNLPhe-100 μM vs. kV/PBS | 2.6 | <0.05 |
| **MV/CuNLPhe-100μM** | 0.034 ± 0.004 | MV/CuNLPhe-100μM vs. MV/PBS | 3.5 | < 0.01 |
