# Supplementary material for: The radioenhancement potential of Schiff base derived copper (II) compounds against lung carcinoma in vitro
Source: PLoS One. 2021 Jun 18;16(6):e0253553. doi: 10.1371/journal.pone.0253553 (PMC8213134; doi:10.1371/journal.pone.0253553)
Supplement: S19 Table — Ctrl/PBS–non-irradiated cells with PBS; kV/PBS–cells with PBS irradiated with 1 Gy at 120 kV; MV/PBS—cells with PBS irradiated with 1 Gy at 6 MV; Ctrl/CuPLTyr-10μM—non-irradiated cells treated with 10 μM Cu(Picolinyl-L-Tyrosinate)2; kV/CuPLTyr-10μM—cells treated with 10 μM Cu(Picolinyl-L-Tyrosinate)2 and irradiated with 1 Gy at 120 kV; MV/CuPLTyr-10μM—cells treated with 10 μM Cu(Picolinyl-L-Tyrosinate)2 and irradiated with 1 Gy at 6 MV; Ctrl/CuPLTyr-100μM—non-irradiated cells treated with 100 μM Cu(Picolinyl-L-Tyrosinate)2; kV/CuPLTyr-100μM—cells treated with 100 μM Cu(Picolinyl-L-Tyrosinate)2 and irradiated with 1 Gy at 120 kV; MV/CuPLTyr-100μM—cells treated with 100 μM Cu(Picolinyl-L-Tyrosinate)2 and irradiated with 1 Gy at 6 MV; M ± SEM–mean ± standard error of the mean. (DOCX) [file pone.0253553.s019.docx]

**S19 Table. Statistical characteristics of the cell count of the HT-29 human colon cancer cells treated with Cu(Picolinyl-L-Tyrosinate)_2._** Ctrl/PBS – non-irradiated cells with PBS; kV/PBS – cells with PBS irradiated with 1 Gy at 120 kV; MV/PBS - cells with PBS irradiated with 1 Gy at 6 MV; Ctrl/CuPLTyr-10μM - non-irradiated cells treated with 10 μM Cu(Picolinyl-L-Tyrosinate)_2_; kV/CuPLTyr-10μM - cells treated with 10 μM Cu(Picolinyl-L-Tyrosinate)_2_ and irradiated with 1 Gy at 120 kV; MV/CuPLTyr-10μM - cells treated with 10 μM Cu(Picolinyl-L-Tyrosinate)_2_ and irradiated with 1 Gy at 6 MV; Ctrl/CuPLTyr-100μM - non-irradiated cells treated with 100 μM Cu(Picolinyl-L-Tyrosinate)_2_; kV/CuPLTyr-100μM - cells treated with 100 μM Cu(Picolinyl-L-Tyrosinate)_2_ and irradiated with 1 Gy at 120 kV; MV/CuPLTyr-100μM - cells treated with 100 μM Cu(Picolinyl-L-Tyrosinate)_2_ and irradiated with 1 Gy at 6 MV; *M ± SEM – mean ± standard error of the mean.*

| **Group** | **Days** | **Мean ± SEM** | **Compared groups** | **Difference (times)** | ***P*** |
| --- | --- | --- | --- | --- | --- |
| **Ctrl/CuPLTyr-10μM** | **Day 8** | 16025 ± 675 | Ctrl/CuPLTyr-10μM vs. Ctrl/PBS | 11.3 | < 0.0001 |
| **kV/CuPLTyr-10μM** | **Day 8** | 10100 ± 500 | kV/CuPLTyr-10μM vs. kV/PBS | 11.4 | < 0.0001 |
| **MV/CuPLTyr-10μM** | **Day 8** | 11450 ± 1650 | MV/CuPLTyr-10μM vs. MV/PBS | 12.7 | < 0.0001 |
| **Ctrl/CuPLTyr-100μM** | **Day 8** | 950 ± 150 | Ctrl/CuPLTyr-100μM vs. Ctrl/PBS | 190.1 | < 0.0001 |
| **kV/CuPLTyr-100μM** | **Day 8** | 5450 ± 450 | kV/CuPLTyr-100μM vs. kV/PBS | 21.2 | < 0.0001 |
| **MV/CuPLTyr-100μM** | **Day 8** | 3875 ± 475 | MV/CuPLTyr-100μM vs. MV/PBS | 37.4 | < 0.0001 |
