# Supplementary material for: The radioenhancement potential of Schiff base derived copper (II) compounds against lung carcinoma in vitro
Source: PLoS One. 2021 Jun 18;16(6):e0253553. doi: 10.1371/journal.pone.0253553 (PMC8213134; doi:10.1371/journal.pone.0253553)
Supplement: S20 Table — Ctrl/PBS–non-irradiated cells with PBS; kV/PBS–cells with PBS irradiated with 1 Gy at 120 kV; MV/PBS—cells with PBS irradiated with 1 Gy at 6 MV; Ctrl/CuILTyr-10μM—non-irradiated cells treated with 10 μM Cu(Isonicotinyl-L-Tyrosinate)2; kV/CuILTyr-10μM—cells treated with 10 μM Cu(Isonicotinyl-L-Tyrosinate)2 and irradiated with 1 Gy at 120 kV; MV/CuILTyr-10μM—cells treated with 10 μM Cu(Isonicotinyl-L-Tyrosinate)2 and irradiated with 1 Gy at 6 MV; Ctrl/CuILTyr-100μM—non-irradiated cells treated with 100 μM Cu(Isonicotinyl-L-Tyrosinate)2; kV/CuILTyr-100μM—cells treated with 100 μM Cu(Isonicotinyl-L-Tyrosinate)2 and irradiated with 1 Gy at 120 kV; MV/CuILTyr-100μM—cells treated with 100 μM Cu(Isonicotinyl-L-Tyrosinate)2 and irradiated with 1 Gy at 6 MV; M ± SEM–mean ± standard error of the mean. (DOCX) [file pone.0253553.s020.docx]

**S20 Table. Statistical characteristics of the cell count of the HT-29 human colon cancer cells treated with Cu(Isonicotinyl-L-Tyrosinate)_2._** Ctrl/PBS – non-irradiated cells with PBS; kV/PBS – cells with PBS irradiated with 1 Gy at 120 kV; MV/PBS - cells with PBS irradiated with 1 Gy at 6 MV; Ctrl/CuILTyr-10μM - non-irradiated cells treated with 10 μM Cu(Isonicotinyl-L-Tyrosinate)_2_; kV/CuILTyr-10μM - cells treated with 10 μM Cu(Isonicotinyl-L-Tyrosinate)_2_ and irradiated with 1 Gy at 120 kV; MV/CuILTyr-10μM - cells treated with 10 μM Cu(Isonicotinyl-L-Tyrosinate)_2_ and irradiated with 1 Gy at 6 MV; Ctrl/CuILTyr-100μM - non-irradiated cells treated with 100 μM Cu(Isonicotinyl-L-Tyrosinate)_2_; kV/CuILTyr-100μM - cells treated with 100 μM Cu(Isonicotinyl-L-Tyrosinate)_2_ and irradiated with 1 Gy at 120 kV; MV/CuILTyr-100μM - cells treated with 100 μM Cu(Isonicotinyl-L-Tyrosinate)_2_ and irradiated with 1 Gy at 6 MV; *M ± SEM – mean ± standard error of the mean*.

| **Group** | **Days** | **Мean ± SEM** | **Compared groups** | **Difference (times)** | ***P*** |
| --- | --- | --- | --- | --- | --- |
| **Ctrl/CuILTyr-10μM** | **Day 8** | 90050 ± 14400 | Ctrl/CuILTyr-10μM vs. Ctrl/PBS | 2.0 | < 0.0001 |
|  |  |  | Ctrl/CuILTyr-10μM vs. Ctrl/CuILTyr-100μM | 4.0 | < 0.0001 |
| **kV/CuILTyr-10μM** | **Day 8** | 72275 ± 13525 | kV/CuILTyr-10μM vs. kV/PBS | 1.6 | < 0.01 |
|  |  |  | kV/CuILTyr-10μM vs. kV/CuILTyr-100μM | 2.5 | < 0.01 |
| **MV/CuILTyr-10μM** | **Day 8** | 99175 ± 1425 | MV/CuILTyr-10μM vs. MV/PBS | 1.5 | < 0.01 |
|  |  |  | MV/CuILTyr-10μM vs. MV/CuILTyr-100μM | 2.1 | < 0.001 |
| **Ctrl/CuILTyr-100μM** | **Day 8** | 22700 ± 2400 | Ctrl/CuILTyr-100μM vs. Ctrl/PBS | 8.0 | < 0.0001 |
| **kV/CuILTyr-100μM** | **Day 8** | 28625 ± 20075 | kV/CuILTyr-100μM vs. kV/PBS | 4.0 | < 0.0001 |
| **MV/CuILTyr-100μM** | **Day 8** | 46900 ± 14150 | MV/CuILTyr-100μM vs. MV/PBS | 3.1 | < 0.0001 |
