# Supplementary material for: The radioenhancement potential of Schiff base derived copper (II) compounds against lung carcinoma in vitro
Source: PLoS One. 2021 Jun 18;16(6):e0253553. doi: 10.1371/journal.pone.0253553 (PMC8213134; doi:10.1371/journal.pone.0253553)
Supplement: S21 Table — Ctrl/PBS–non-irradiated cells with PBS; kV/PBS–cells with PBS irradiated with 1 Gy at 120 kV; MV/PBS—cells with PBS irradiated with 1 Gy at 6 MV; Ctrl/CuPLTrp-10μM—non-irradiated cells treated with 10 μM Cu(Picolinyl-L-Tryptophanate)2; kV/CuPLTrp-10μM—cells treated with 10 μM Cu(Picolinyl-L-Tryptophanate)2 and irradiated with 1 Gy at 120 kV; MV/CuPLTrp-10μM—cells treated with 10 μM Cu(Picolinyl-L-Tryptophanate)2 and irradiated with 1 Gy at 6 MV; Ctrl/CuPLTrp-100μM—non-irradiated cells treated with 100 μM Cu(Picolinyl-L-Tryptophanate)2; kV/CuPLTrp-100μM—cells treated with 100 μM Cu(Picolinyl-L-Tryptophanate)2 and irradiated with 1 Gy at 120 kV; MV/CuPLTrp-100μM—cells treated with 100 μM Cu(Picolinyl-L-Tryptophanate)2 and irradiated with 1 Gy at 6 MV; M ± SEM–mean ± standard error of the mean. (DOCX) [file pone.0253553.s021.docx]

**S21 Table. Statistical characteristics of the cell count of the HT-29 human colon cancer cells treated with Cu(Picolinyl-L-Tryptophanate)_2._** Ctrl/PBS – non-irradiated cells with PBS; kV/PBS – cells with PBS irradiated with 1 Gy at 120 kV; MV/PBS - cells with PBS irradiated with 1 Gy at 6 MV; Ctrl/CuPLTrp-10μM - non-irradiated cells treated with 10 μM Cu(Picolinyl-L-Tryptophanate)_2_; kV/CuPLTrp-10μM - cells treated with 10 μM Cu(Picolinyl-L-Tryptophanate)_2_ and irradiated with 1 Gy at 120 kV; MV/CuPLTrp-10μM - cells treated with 10 μM Cu(Picolinyl-L-Tryptophanate)_2_ and irradiated with 1 Gy at 6 MV; Ctrl/CuPLTrp-100μM - non-irradiated cells treated with 100 μM Cu(Picolinyl-L-Tryptophanate)_2_; kV/CuPLTrp-100μM - cells treated with 100 μM Cu(Picolinyl-L-Tryptophanate)_2_ and irradiated with 1 Gy at 120 kV; MV/CuPLTrp-100μM - cells treated with 100 μM Cu(Picolinyl-L-Tryptophanate)_2_ and irradiated with 1 Gy at 6 MV; *M ± SEM – mean ± standard error of the mean*.

| **Group** | **Days** | **Мean ± SEM** | **Compared groups** | **Difference (times)** | ***P*** |
| --- | --- | --- | --- | --- | --- |
| **Ctrl/CuPLTrp-10μM** | **Day 8** | 64975 ± 1675 | Ctrl/CuPLTrp-10μM vs. Ctrl/PBS | 2.8 | < 0.0001 |
|  |  |  | Ctrl/CuPLTrp-10μM vs. MV/CuPLTrp-10μM | 2.5 | < 0.0001 |
|  |  |  | Ctrl/CuPLTrp-10μM vs. Ctrl/CuPLTrp-100μM | 13.2 | < 0.0001 |
| **kV/CuPLTrp-10μM** | **Day 8** | 42875 ± 6175 | kV/CuPLTrp-10μM vs. kV/PBS | 2.7 | <0.0001 |
|  |  |  | kV/CuPLTrp-10μM vs. kV/CuPLTrp-100μM | 7.2 | <0.001 |
| **MV/CuPLTrp-10μM** | **Day 8** | 26475 ± 4125 | MV/CuPLTrp-10μM vs. MV/PBS | 5.5 | < 0.0001 |
| **Ctrl/CuPLTrp-100μM** | **Day 8** | 4925 ± 1375 | Ctrl/CuPLTrp-100μM vs. Ctrl/PBS | 36.7 | < 0.0001 |
| **kV/CuPLTrp-100μM** | **Day 8** | 5925 ± 575 | kV/CuPLTrp-100μM vs. kV/PBS | 19.5 | <0.0001 |
| **MV/CuPLTrp-100μM** | **Day 8** | 4050 ± 700 | MV/CuPLTrp-100μM vs. MV/PBS | 35.8 | < 0.0001 |
