# Supplementary material for: The radioenhancement potential of Schiff base derived copper (II) compounds against lung carcinoma in vitro
Source: PLoS One. 2021 Jun 18;16(6):e0253553. doi: 10.1371/journal.pone.0253553 (PMC8213134; doi:10.1371/journal.pone.0253553)
Supplement: S22 Table — Ctrl/PBS–non-irradiated cells with PBS; kV/PBS–cells with PBS irradiated with 1 Gy at 120 kV; MV/PBS—cells with PBS irradiated with 1 Gy at 6 MV; Ctrl/CuPLPhe-10μM—non-irradiated cells treated with 10 μM Cu(Picolinyl-L- Phenylalaninate)2; kV/CuPLPhe-10μM—cells treated with 10 μM Cu(Picolinyl-L- Phenylalaninate)2 and irradiated with 1 Gy at 120 kV; MV/CuPLPhe-10μM—cells treated with 10 μM Cu(Picolinyl-L- Phenylalaninate)2 and irradiated with 1 Gy at 6 MV; Ctrl/CuPLPhe-100μM—non-irradiated cells treated with 100 μM Cu(Picolinyl-L- Phenylalaninate)2; kV/CuPLPhe-100μM—cells treated with 100 μM Cu(Picolinyl-L- Phenylalaninate)2 and irradiated with 1 Gy at 120 kV; MV/CuPLPhe-100μM—cells treated with 100 μM Cu(Picolinyl-L-Phenylalaninate)2 and irradiated with 1 Gy at 6 MV; M ± SEM–mean ± standard error of the mean. (DOCX) [file pone.0253553.s022.docx]

**S22 Table. Statistical characteristics of the cell count of the HT-29 human colon cancer cells treated with Cu(Picolinyl-L-Phenylalaninate)_2._** Ctrl/PBS – non-irradiated cells with PBS; kV/PBS – cells with PBS irradiated with 1 Gy at 120 kV; MV/PBS - cells with PBS irradiated with 1 Gy at 6 MV; Ctrl/CuPLPhe-10μM - non-irradiated cells treated with 10 μM Cu(Picolinyl-L- Phenylalaninate)_2_; kV/CuPLPhe-10μM - cells treated with 10 μM Cu(Picolinyl-L- Phenylalaninate)_2_ and irradiated with 1 Gy at 120 kV; MV/CuPLPhe-10μM - cells treated with 10 μM Cu(Picolinyl-L- Phenylalaninate)_2_ and irradiated with 1 Gy at 6 MV; Ctrl/CuPLPhe-100μM - non-irradiated cells treated with 100 μM Cu(Picolinyl-L- Phenylalaninate)_2_; kV/CuPLPhe-100μM - cells treated with 100 μM Cu(Picolinyl-L- Phenylalaninate)_2_ and irradiated with 1 Gy at 120 kV; MV/CuPLPhe-100μM - cells treated with 100 μM Cu(Picolinyl-L-Phenylalaninate)_2_ and irradiated with 1 Gy at 6 MV; *M ± SEM – mean ± standard error of the mean*.

| **Group** | **Days** | **Мean ± SEM** | **Compared groups** | **Difference (times)** | ***P*** |
| --- | --- | --- | --- | --- | --- |
| **Ctrl/CuPLPhe-10μM** | **Day 8** | 4600 ± 1150 | Ctrl/CuPLPhe-10μM vs. Ctrl/PBS | 39.3 | < 0.0001 |
| **kV/CuPLPhe-10μM** | **Day 8** | 4350 ± 750 | kV/CuPLPhe-10μM vs. kV/PBS | 26.5 | < 0.0001 |
| **MV/CuPLPhe-10μM** | **Day 8** | 6025 ± 775 | MV/CuPLPhe-10μM vs. MV/PBS | 24.1 | < 0.0001 |
| **Ctrl/CuPLPhe-100μM** | **Day 8** | 1175 ± 275 | Ctrl/CuPLPhe-100μM vs. Ctrl/PBS | 153.7 | < 0.0001 |
| **kV/CuPLPhe-100μM** | **Day 8** | 1525 ± 125 | kV/CuPLPhe-100μM vs. kV/PBS | 75.6 | < 0.0001 |
| **MV/CuPLPhe-100μM** | **Day 8** | 2625 ± 175 | MV/CuPLPhe-100μM vs. MV/PBS | 55.3 | < 0.0001 |
