# Supplementary material for: The radioenhancement potential of Schiff base derived copper (II) compounds against lung carcinoma in vitro
Source: PLoS One. 2021 Jun 18;16(6):e0253553. doi: 10.1371/journal.pone.0253553 (PMC8213134; doi:10.1371/journal.pone.0253553)
Supplement: S23 Table — Ctrl/PBS–non-irradiated cells with PBS; MV/PBS—cells with PBS irradiated with 1 Gy at 6 MV; Ctrl/CuNLPhe-10μM—non-irradiated cells treated with 10 μM Cu(Nicotinyl-L- Phenylalaninate)2; kV/CuNLPhe-10μM—cells treated with 10 μM Cu(Nicotinyl-L- Phenylalaninate)2 and irradiated with 1Gy at 120 kV; MV/CuNLPhe-10μM—cells treated with 10 μM Cu(Nicotinyl-L- Phenylalaninate)2 and irradiated with 1Gy at 6 MV; Ctrl/CuNLPhe-100μM—non-irradiated cells treated with 100 μM Cu(Nicotinyl-L- Phenylalaninate)2; MV/CuNLPhe-100μM—cells treated with 100 μM Cu(Nicotinyl-L-Phenylalaninate)2 and irradiated with 1 Gy at 6 MV; M ± SEM–mean ± standard error of the mean. (DOCX) [file pone.0253553.s023.docx]

**S23 Table. Statistical characteristics of the cell count of the HT-29 human colon cancer cells treated with Cu(Nicotinyl-L-Phenylalaninate)_2._** Ctrl/PBS – non-irradiated cells with PBS; MV/PBS - cells with PBS irradiated with 1 Gy at 6 MV; Ctrl/CuNLPhe-10μM - non-irradiated cells treated with 10 μM Cu(Nicotinyl-L- Phenylalaninate)_2_; kV/CuNLPhe-10μM - cells treated with 10 μM Cu(Nicotinyl-L- Phenylalaninate)_2_ and irradiated with 1Gy at 120 kV; MV/CuNLPhe-10μM - cells treated with 10 μM Cu(Nicotinyl-L- Phenylalaninate)_2_ and irradiated with 1Gy at 6 MV; Ctrl/CuNLPhe-100μM - non-irradiated cells treated with 100 μM Cu(Nicotinyl-L- Phenylalaninate)_2_; MV/CuNLPhe-100μM - cells treated with 100 μM Cu(Nicotinyl-L-Phenylalaninate)_2_ and irradiated with 1 Gy at 6 MV; *M ± SEM – mean ± standard error of the mean*.

| **Group** | **Days** | **Мean ± SEM** | **Compared groups** | **Difference (times)** | ***P*** |
| --- | --- | --- | --- | --- | --- |
| **Ctrl/CuNLPhe-10μM** | **Day 8** | 113425 ± 16925 | Ctrl/CuNLPhe-10μM vs. Ctrl/PBS | 1.6 | < 0.0001 |
| **kV/CuNLPhe-10μM** | **Day 8** | 126000 ± 2250 | kV/CuNLPhe-10μM vs. MV/CuNLPhe-10μM | 1.7 | < 0.0001 |
| **MV/CuNLPhe-10μM** | **Day 8** | 76575 ± 19875 | MV/CuNLPhe-10μM vs. MV/PBS | 1.9 | < 0.0001 |
| **Ctrl/CuNLPhe-100μM** | **Day 8** | 77025 ± 13275 | Ctrl/CuNLPhe-100μM vs. Ctrl/PBS | 2.3 | < 0.0001 |
| **MV/CuNLPhe-100μM** | **Day 8** | 79800 ± 8700 | MV/CuNLPhe-100μM vs. MV/PBS | 1.8 | < 0.0001 |
