# Supplementary material for: The radioenhancement potential of Schiff base derived copper (II) compounds against lung carcinoma in vitro
Source: PLoS One. 2021 Jun 18;16(6):e0253553. doi: 10.1371/journal.pone.0253553 (PMC8213134; doi:10.1371/journal.pone.0253553)
Supplement: S24 Table — Ctrl/PBS–non-irradiated cells only with PBS; kV/PBS–cells with PBS irradiated with 1 Gy at 120 kV; MV/PBS–cells with PBS irradiated with 1 Gy at 6 MV; Ctrl/CuILPhe-10μM—non-irradiated cells treated with 10 μM Cu(Isonicotinyl-L- Phenylalaninate)2; kV/CuILPhe-10μM—cells treated with 10 μM Cu(Isonicotinyl-L- Phenylalaninate)2 and irradiated with 1 Gy at 120 kV; MV/CuILPhe-10μM—cells treated with 10 μM Cu(Isonicotinyl-L- Phenylalaninate)2 and irradiated with 1 Gy at 6 MV; Ctrl/CuILPhe-100μM—non-irradiated cells treated with 100 μM Cu(Isonicotinyl-L- Phenylalaninate)2; kV/CuILPhe-100μM—cells treated with 100 μM Cu(Isonicotinyl-L- Phenylalaninate)2 and irradiated with 1 Gy at 120 kV; MV/CuILPhe-100μM—cells treated with 100 μM Cu(Isonicotinyl-L-Phenylalaninate)2 and irradiated with 1 Gy at 10 MV; M ± SEM–mean ± standard error of the mean. (DOCX) [file pone.0253553.s024.docx]

**S24 Table. Statistical characteristics of the cell count of the HT-29 human colon cancer cells treated with Cu(Isonicotinyl-L-Phenylalaninate)_2._** Ctrl/PBS – non-irradiated cells only with PBS; kV/PBS – cells with PBS irradiated with 1 Gy at 120 kV; MV/PBS – cells with PBS irradiated with 1 Gy at 6 MV; Ctrl/CuILPhe-10μM - non-irradiated cells treated with 10 μM Cu(Isonicotinyl-L- Phenylalaninate)_2_; kV/CuILPhe-10μM - cells treated with 10 μM Cu(Isonicotinyl-L- Phenylalaninate)_2_ and irradiated with 1 Gy at 120 kV; MV/CuILPhe-10μM - cells treated with 10 μM Cu(Isonicotinyl-L- Phenylalaninate)_2_ and irradiated with 1 Gy at 6 MV; Ctrl/CuILPhe-100μM - non-irradiated cells treated with 100 μM Cu(Isonicotinyl-L- Phenylalaninate)_2_; kV/CuILPhe-100μM - cells treated with 100 μM Cu(Isonicotinyl-L- Phenylalaninate)_2_ and irradiated with 1 Gy at 120 kV; MV/CuILPhe-100μM - cells treated with 100 μM Cu(Isonicotinyl-L-Phenylalaninate)_2_ and irradiated with 1 Gy at 10 MV; *M ± SEM – mean ± standard error of the mean*.

| **Group** | **Days** | **Мean ± SEM** | **Compared groups** | **Difference (times)** | ***P*** |
| --- | --- | --- | --- | --- | --- |
| **Ctrl/CuILPhe-10μM** | **Day 8** | 113175 ± 11825 | Ctrl/CuILPhe-10μM vs. Ctrl/PBS | 1.6 | < 0.0001 |
|  |  |  | Ctrl/CuILPhe-10μM vs. kV/CuILPhe-10μM | 1.4 | < 0.01 |
|  |  |  | Ctrl/CuILPhe-10μM vs. MV/CuILPhe-10μM | 1.5 | < 0.05 |
| **kV/CuILPhe-10μM** | **Day 8** | 157175 ± 23675 | kV/CuILPhe-10μM vs. kV/PBS | 1.4 | < 0.01 |
|  |  |  | kV/CuILPhe-10μM vs. MV/CuILPhe-10μM | 2.1 | < 0.0001 |
|  |  |  | kV/CuILPhe-10μM vs. kV/CuILPhe-100μM | 1.5 | < 0.001 |
| **MV/CuILPhe-10μM** | **Day 8** | 76350 ± 8950 | MV/CuILPhe-10μM vs. MV/PBS | 1.9 | < 0.0001 |
| **Ctrl/CuILPhe-100μM** | **Day 8** | 83500 ± 750 | Ctrl/CuILPhe-100μM vs. Ctrl/PBS | 2.2 | < 0.0001 |
|  |  |  | Ctrl/CuILPhe-100μM vs. MV/CuILPhe-100μM | 1.3 | < 0.01 |
| **kV/CuILPhe-100μM** | **Day 8** | 104875 ± 7725 | kV/CuILPhe-100μM vs. MV/CuILPhe-100μM | 2.5 | < 0.0001 |
| **MV/CuILPhe-100μM** | **Day 8** | 41250 ± 2600 | MV/CuILPhe-100μM vs. MV/PBS | 3.5 | < 0.0001 |
